# Thermally Degradable Biocompatible Hydrogel as Transient Encapsulation Coating for Implantable Sensors

**DOI:** 10.1002/advs.202517202

**Published:** 2026-04-16

**Authors:** Francesca Persano, Eleonora Vandini, Eleonora Daini, Antonietta Vilella, Daniela Giuliani, Leonardo Lamanna, Marco Friuli, Christian Demitri, Laura Pecoraro, Amilcare Barca, Tiziano Verri, Cosimino Malitesta, Martina Corsi, Giuseppe Barillaro, Elisabetta Mazzotta

**Affiliations:** ^1^ Laboratory of Analytical Chemistry Department of Biological and Environmental Sciences and Technologies (Di.S.Te.B.A.) University of Salento Lecce Italy; ^2^ Department of Biomedical, Metabolic and Neural Sciences University of Modena and Reggio Emilia via G. Campi 287 Modena Italy; ^3^ Department of Engineering for Innovation Campus Ecotekne University of Salento Lecce Italy; ^4^ Department of Experimental Medicine University of Salento Lecce Italy; ^5^ Laboratory of Applied Physiology Department of Biological and Environmental Sciences and Technologies (Di.S.Te.B.A.) University of Salento Lecce Italy; ^6^ Laboratory of Applied Physiology Department of Experimental Medicine (Di.Me.S.) University of Salento Lecce Italy; ^7^ Department of Information Engineering University of Pisa via G. Caruso 16 Pisa Italy

**Keywords:** biodegradable device, implantable sensor, protective coating, stimuli‐responsive material, thermo‐reversible encapsulating coating

## Abstract

Implantable sensors are redefining real‐time, continuous physiological monitoring and hold great promise for personalized medicine. Yet, their clinical application is often hindered by the lack of effective encapsulation materials that simultaneously ensure environmental exposure and protect sensor components, thereby preserving signal fidelity. Although hydrogel‐based systems offer promising features such as biological tissue‐like mechanics and biocompatibility, their use as sensor coatings remains limited. Notably, despite growing interest in bioresorbable implant technologies, few coating systems offer controlled, on‐demand degradability, which represents a key requirement to avoid possible misalignment between coating dissolution rate and sensor lifetime. Here, we present a thermoresponsive hydrogel, consisting of methylcellulose, polyethylene glycol and polyacrylic acid (MC/PEG/PAA), demonstrating its high suitability as a transient encapsulating coating. The system, assembled by physical cross‐linking without using toxic reagents, and extensively characterized in terms of thermal responsiveness, swelling behavior, stability, and mechanical performance, exhibited reversible dissolution upon a mild temperature drop (from ≈37°C to ≈25°C), which was conveniently used to easily enable its degradation under physiological conditions. The hydrogel ability to act as a coating was demonstrated via the encapsulation of a fluorescent model device, confirming its optical transparency and signal‐transmission capability. In vitro studies indicated the hydrogel potential to mitigate inflammatory response, while in vivo biocompatibility, assessed in mice, revealed no signs of systemic toxicity or behavioral changes post‐implantation. Additionally, the hydrogel exhibited safe in vivo biodegradation upon a slight local temperature decrease, completely dissolving within few minutes. The developed coating, integrating good optical transparency, ease of fabrication, and in vivo safety with on‐demand, controllable degradation upon an external low‐temperature stimulus, represents a substantial advancement toward next‐generation bioresorbable implantable devices and paves the way for driving successful transformation in the field.

## Graphical abstract

A biocompatible hydrogel coating with tissue‐like mechanics and reversible dissolution triggered by a mild, physiologically compatible temperature drop (≈37°C → ≈25°C) was developed for the transient encapsulation of implantable devices. The physically crosslinked methylcellulose, polyethylene glycol and polyacrilic acid (MC/PEG/PAA) hydrogel effectively protects the device while maintaining optical transparency and signal transmission. In vitro, the coating reduced fibroblast adhesion and showed anti‐inflammatory potential. In vivo studies in mice confirmed complete, safe degradation upon local cooling, with no systemic toxicity or behavioral alterations. This controllable, on‐demand dissolution strategy marks a significant step toward next‐generation bioresorbable implantable systems.

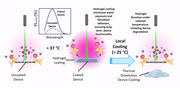

## 1 Introduction

Implantable sensors have revolutionized the way of monitoring biophysical and biochemical parameters within the body by enabling real‐time interventions and timely adjustments to treatment plans, representing a significant step ahead in the field of precision diagnostics and personalized medicine [1]. To ensure reliable and durable operation of biointegrated implantable devices, some key issues possibly degrading their performance must be critically considered. First, their long‐term functionality can be limited by adverse immune responses and biofouling [2, 3, 4, 5]. Upon the implantation of the device, an inflammatory response of the body can occur, triggered by the early adsorption of plasma proteins [6, 7], which activates neutrophils, macrophages, and fibroblasts [8, 9, 10], leading to the formation of a dense fibrotic capsule [11, 12] that hinders molecules diffusion and compromises sensor sensitivity [13, 14]. In addition to immunogenicity, the mechanical mismatch between rigid device materials and soft tissues can induce chronic microtrauma, aggravating local inflammation and accelerating device rejection from the target tissues [15, 16]. Furthermore, during long‐term operation, the dynamic nature of the biological tissue can determine volume changes, resulting in mechanical stress that the device must accommodate to maintain stability. Finally, phenomena such as leaching of functional materials and moisture absorption can contribute to device signal drift over time.

Encapsulation layers are commonly applied to safeguard the functional components of implantable devices. While insulating polymeric materials such as silicone, polyurethane, and parylene are among the most frequently used, serving as effective barriers against moisture and mechanical stress [17, 18], hydrogel‐based coatings have more recently emerged as surface protection layers, helping to prevent the attachment of cells and biomolecules, thereby improving anti‐biofouling performance. Valuable features in such applications also include their high biocompatibility and mechanical softness [19, 20, 21, 22, 23]. Hydrogels with tissue‐like elastic properties and high fracture toughness offer indeed a more compliant interface, capable of adapting to the dynamic biological environment. Leveraging a Young's modulus closely matching that of biological tissues, hydrogels revealed to effectively reduce the mechanical mismatch at the biointerface and minimize immune response [24, 25].

Despite substantial progress in the design of hydrogels for general biomedical implants, their application to sensing devices remains relatively underexplored. This may be ascribed to key additional functions that the hydrogels must fulfil in this case, ensuring the continuous access of the target analytes to the device sensitive surface [16], while providing a balance between safeguarding of the device during its operation and transmitting an unattenuated stable signal outside the body. In this sense, hydrogels with high transparency and good conductivity are highly desirable as encapsulation layers of optical and electrochemical sensors, respectively, which are the most applied in the field of implantable monitoring [26].

Examples of hydrogels developed as coating layers of sensors include hydroxyethyl methacrylate (HEMA) cross‐linked with ethylene glycol dimethacrylate (EGDMA) and N‐vinyl‐2‐pyrrolidone (VP) [27], agarose [28], polyethylene glycol diacrylate (PEGDA) and acrylamide‐based hydrogels [29], mainly selected for their ability to provide minimal signal drift. Nonetheless, their clinical transability was limited by the need for complex synthetic routes requiring laborious purification protocols and/or toxic reagents and/or UV‐induced crosslinking, possibly compromising sensor biocomponents, along with the lack of evaluation of long‐term performance.

Additionally, in light of the ongoing revolution in the field of implantable sensors – driven by advances in bioresorbable sensor technologies – encapsulating layers should be designed and engineered accordingly, to remain aligned with the technological advancements in the field. Bioresorbable sensors are indeed redefining precision medicine, enabling to avoid surgical removal, with valuable benefits in terms of reduced costs and complications. So far, hydrogels such as polylactic acid (PLA) [30, 31, 32] and poly(lactic‐co‐glycolic acid) (PLGA) [33, 34], along with natural polymers [35, 36, 37, 38] and inorganic dielectric materials [39, 40, 41], have been successfully proposed to this aim, due to their tunable degradation rates, mechanical flexibility and water impermeability. Nonetheless, the possible misalignment between coating dissolution rate and required sensor lifetime poses the risk of not having the encapsulation layer intact until the device has fulfilled its intended function. This paves the way for further substantial progress in the development of these materials leading to smart coating layers degraded upon responding to an external stimulus, thus triggering the on‐demand, controllable degradation of the coated device. Among the few strategies reported so far, promising approaches include UV‐responsive hydrogel–oxide bilayers enabling light‐induced gel‐to‐sol transitions for device disintegration [42], thermally activated wax matrices loaded with acidic microdroplets for rapid thermally triggered degradation [43], and near‐infrared (NIR)‐induced depolymerization of cyclic poly(phthalaldehyde) (cPPA) via photothermal conversion [44]. A major limitation common to these studies, however, is the absence of biocompatibility evaluation, which significantly limits their translational potential for in vivo applications.

To address this critical gap, we developed a novel encapsulating hydrogel with on‐demand degradation through a thermal stimulus – simply consisting of a temperature decrease from body (≈37°C) to room temperature (≈25°C) – enabling gel‐sol transition and consequent hydrogel dissolution. The hydrogel, combining methyl cellulose, polyethylene glycol and polyacrylic acid (MC/PEG/PAA), presents indeed reversible thermo‐responsivity for which, upon gelation at physiological temperature, the sol state is reversibly achieved by simply reducing the temperature to about 25°C. When applied as a coating of bioresorbable systems, just a few degrees of local temperature decrease enables the dissolution of the encapsulating layer with the subsequent exposure of the device to the biofluids, thus overcoming the possible mismatch between the device/coating degradation kinetics. Functional validation using a fluorescent device under physiological conditions confirmed its protective effect in maintaining signal integrity, while cell adhesion assays and in vivo biocompatibility substantiated its high potential as a coating for bioimplant applications. Remarkably, the reversible transition to the sol state, with hydrogel dissolution, was verified in vivo, unequivocally demonstrating the high suitability of the developed smart, transient coating for next‐generation implantable devices.

## 2 Results and Discussion

**FIGURE 1:**
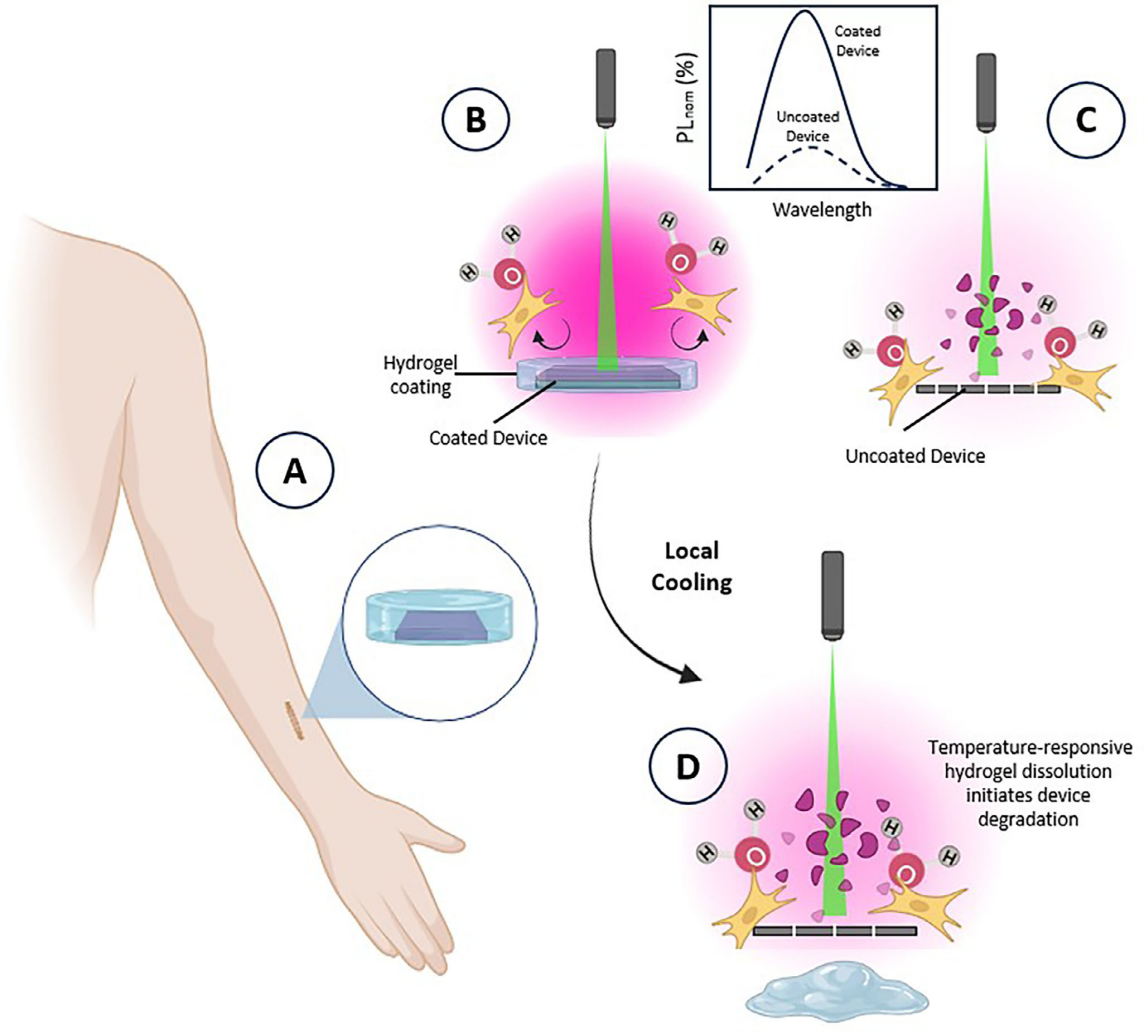
Conceptual schematic illustrating the application of a stimuli‐responsive, thermoreversible hydrogel coating for an implantable fluorescent device. (A) The illustration depicts a model sensor encapsulated within a thermoresponsive hydrogel and implanted subcutaneously in human tissue. (B) Under physiological conditions (37°C), the hydrogel remains optically transparent and structurally stable, effectively shielding the sensor while maintaining its functional performance over time. The coating minimizes water exposure and prevents fibroblast adhesion, both of which could otherwise degrade sensor performance during long‐term implantation, as in the case of the uncoated device (C). (D) Upon localized external cooling, by simply decreasing the temperature to about 25°C, the hydrogel undergoes controlled dissolution, exposing the sensor to the surrounding biological fluids and enabling direct interaction with the tissue microenvironment. This feature can be exploited to trigger the degradation of the biodegradable encapsulated device without the need for surgical removal.

The schematic of the developed encapsulating system is illustrated in Figure 1. The hydrogel embedding the sensor device is intended to be subcutaneously implanted within human tissue (Figure 1A). Under physiological temperature (37°C), the hydrogel remains optically transparent and mechanically stable, providing effective protection for the sensor while preserving its functional integrity over extended implantation periods (Figure 1B). Specifically, the coating limits water exposure and inhibits fibroblast adhesion, thus preventing performance degradation during long‐term operation, which occurs instead in the uncoated device (Figure 1C). Upon localized external cooling to approximately 25°C, the hydrogel undergoes a controlled phase transition (Figure 1D), resulting in its dissolution and subsequent exposure of the sensor to the surrounding biofluids. This thermally responsive behavior enables on‐demand degradation of the biodegradable encapsulated device, thereby obviating the need for surgical removal and overcoming the possible mismatch between the device/coating degradation kinetics.

The hydrogel is assembled by combining methylcellulose and polyethylene glycol (MC/PEG) [45], with three variants incorporating, respectively, PEG diacrylate (PEGDA), PEG methyl ether acrylate (PEGMA), and polyacrylic acid (PAA), added to enhance hydrolytic resistance and modulate water uptake [46, 47, 48], vital for preserving moisture‐sensitive components. A systematic investigation of the four tested systems – aimed at testing their thermoreversibile behavior, mechanical, swelling/wettability properties, and stability under simulated physiological conditions – allowed us to select MC/PEG/PAA as the optimal hydrogel composition.

**FIGURE 2:**
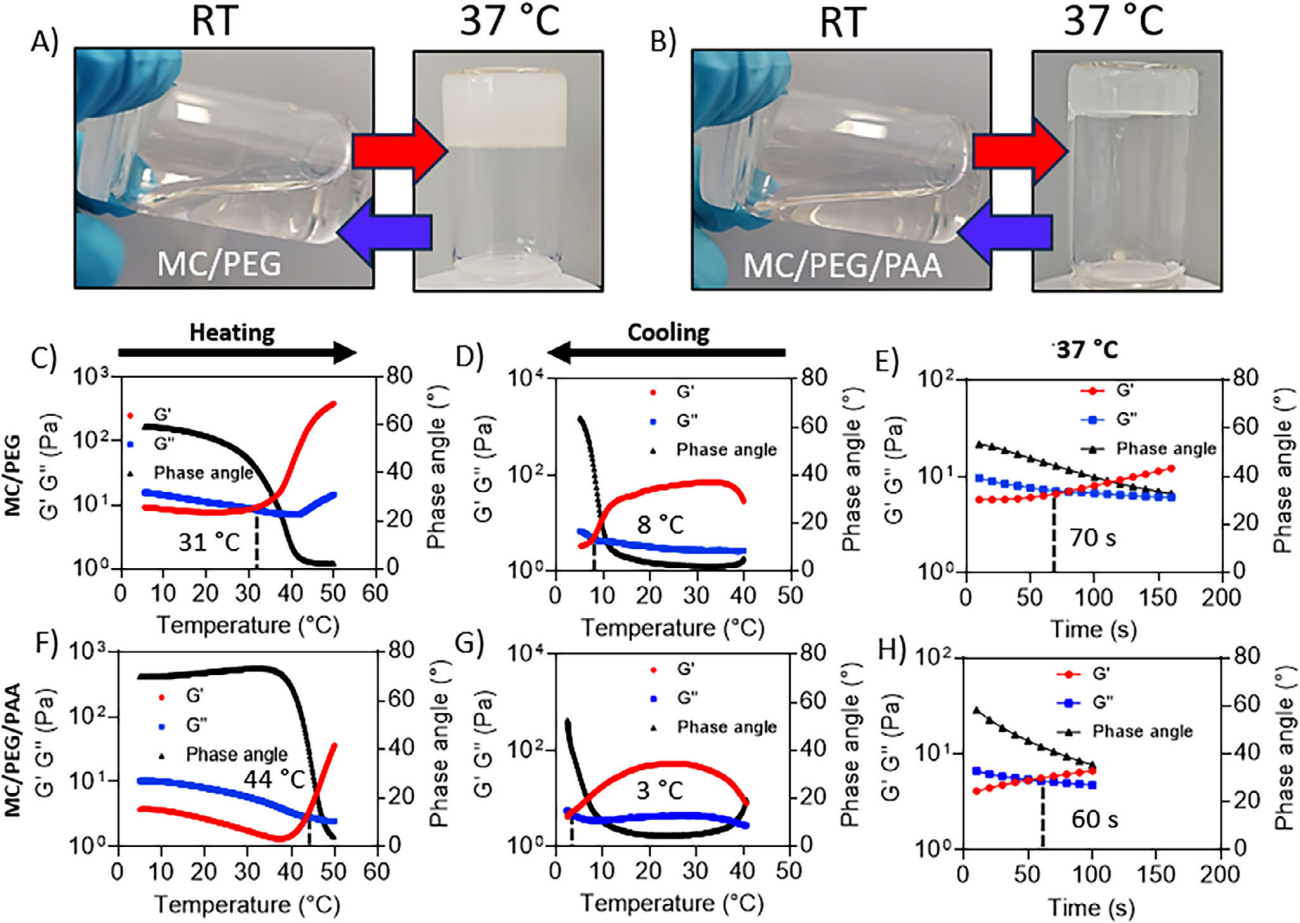
Temperature‐dependent behavior of the hydrogels. (A,B) Inversion tube test on the MC/PEG (A) and MC/PEG/PAA (B). Pictures are taken immediately after the mixing (left) and after incubation at 37°C (right). (C‐H) Rheological analysis of MC/PEG and MC/PEG/PAA hydrogels. (C,F) Samples were heated from 5°C to 50°C at 1°C/min. Frequency was 1 Hz and shear strain 3%. Tgel is defined as the crossover of storage G‵ and loss modulus G‵‵. (D,G) Temperature ramps of gel‐sol transition of MC/PEG and MC/PEG/PAA formulations. Initially, the samples were in the gel state at 37°C and were cooled from 40°C to 5 (or 2.5)°C at 1°C/min. Frequency was 1 Hz and shear strain 3%. T gel‐sol transition is defined as the crossover of storage G‵ and loss modulus G‵‵. (E,H) Gelation kinetics of MC/PEG (E) and MC/PEG/PAA (H) hydrogels at 37°C.

The thermo‐responsiveness of the materials was first evaluated by tube inversion test (Figure 2A,B; Figure S1A,B), clearly showing in all cases the thermo‐reversible sol‐gel transition, enabled by the physical cross‐linking, governed by non‐covalent interactions and susceptible to the external temperature. Rheological studies revealed the same (Figure 2C–H; Figure S1C–F), allowing to estimate the gelation temperature (Tgel) and the melting temperature (Tm) of each hydrogel, during heating and cooling processes, respectively (Table S1). It is evident that PAA (Figure 2F) caused an increase of the gelation point by approximately 13°C compared to MC/PEG alone (Figure 2C), and a decrease of the melting point by about 5°C (Figure 2D–G). This behavior reflects the competing effects introduced by PAA within the MC/PEG network. PAA provides a high density of carboxylic groups capable of forming hydrogen bonds [49] with the hydroxyl groups of methylcellulose (MC) and the ether oxygens of polyethylene glycol (PEG), while simultaneously introducing steric hindrance [50, 51] and extensive hydration shells around the polymer chains. The hydrogen‐bonding capacity of PAA favors polymer–polymer interactions, yet its bulky, hydrated structure interferes with the close packing of MC chains and delays the hydrophobic association responsible for thermal gelation. As a result, a higher temperature is required to overcome these hydration barriers and trigger phase separation, leading to the observed increase in the gelation point. Once gelation occurs, however, the hydrogen‐bonding network among PAA, MC, and PEG contributes to a more cohesive and stable structure. These secondary interactions promote tighter chain association, which lowers the melting temperature. Interestingly, a rapid gelation was observed for the MC/PEG/PAA hydrogel at 37°C (Figure 2H; Figure S2A). This behavior can be attributed to the cooperative interplay between hydrophobic interactions of MC, hydrogen bonding, and the steric and crowding effects of highly hydrated PAA chains. Upon reaching the critical temperature, partial dehydration of MC methoxy groups triggers phase separation, while the bulky and hydrated PAA chains restrict the available volume and promote closer packing of MC chains. This macromolecular crowding facilitates rapid polymer chain rearrangement, resulting in the formation of a compact gel within 60 s, highlighting the dynamic adaptability and fast gelation kinetics of the MC/PEG/PAA system. A different thermo‐responsive behavior is observed for PEGDA‐ and PEGMA‐modified systems (Figure S1C–F), which exhibit a predominantly viscous response up to approximately 20°C, without a clear sol–gel transition, evidenced by the lack of a distinct G′–G″ crossover in the rheological profiles [52, 53]. Although also in these systems, hydrogen bonding between the hydroxyl groups of MC and the acrylate moieties of PEGDA/PEGMA plays a role in the hydrogel structure, it is characterized by an increased compactness with restricted chain mobility, resulting in a dampened thermal transition.

Based on these results, the viscoelastic behavior of MC/PEG/PAA system across increasing strain levels was assessed to evaluate its suitability as a mechanically stable coating, by an amplitude sweep test at 37°C. The results (Figure S2B) confirmed a predominantly elastic profile (G′ > G″) throughout the entire strain range tested, with a phase angle consistently below 10°, suggesting the formulation maintains network integrity even under large deformations. Notably, G′ remained nearly constant up to ≈1% strain, indicating the presence of a well‐defined linear viscoelastic region (LVER), beyond which a slight increase in G′ was observed, likely due to network reinforcement via strain‐induced alignment. The absence of a sharp decline in G′ or an inversion with G″ across strains exceeding 10% underscores the hydrogel mechanical resilience and structural robustness, key attributes for coatings subjected to physiological micromotions and mechanical stress in vivo.

**FIGURE 3:**
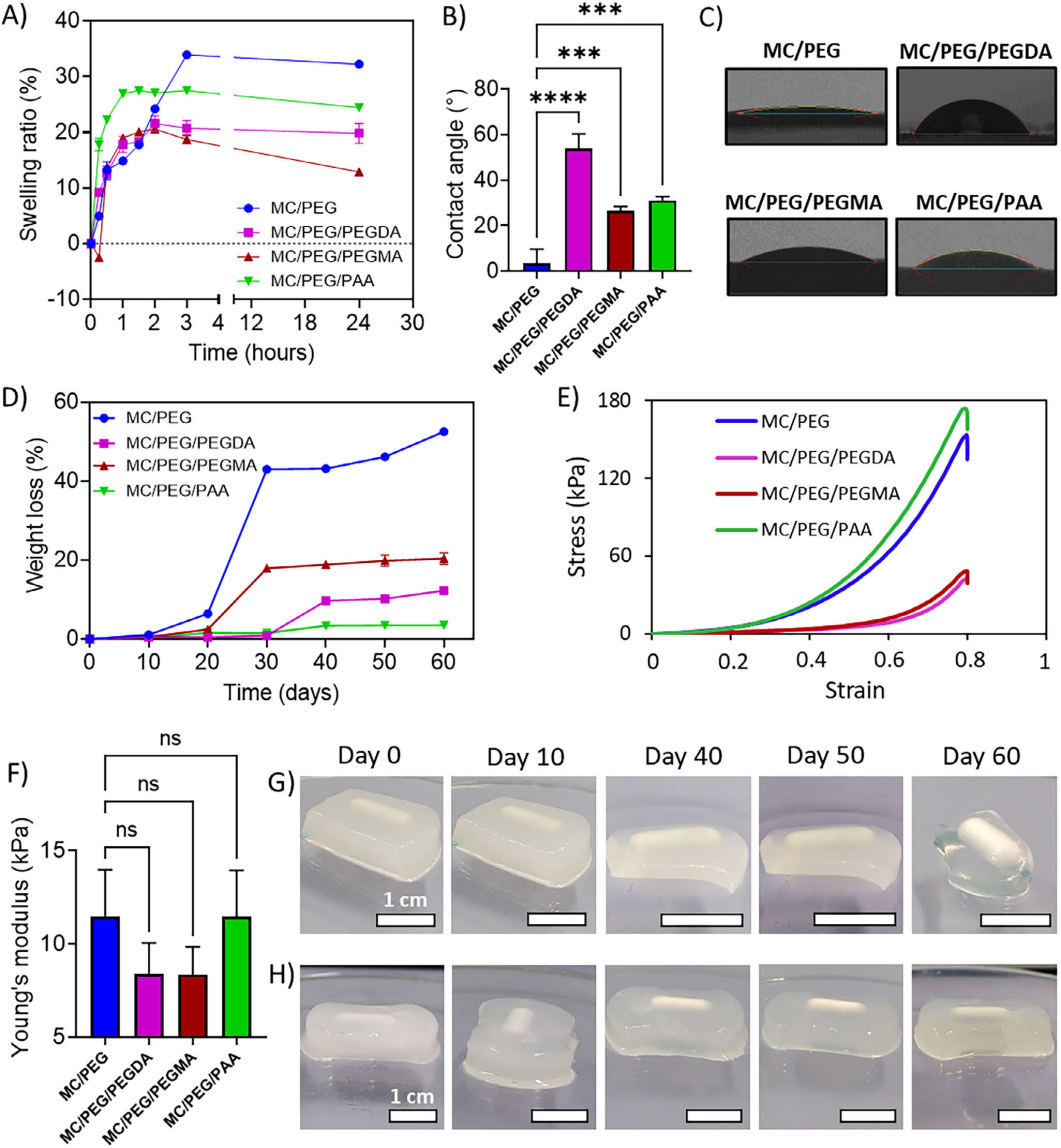
Swelling, wettability, stability tests and mechanical properties of the tested hydrogels. (A) Swelling ratio of hydrogels as a function of time at 37°C. Each point represents the mean ± SD (*n* = 3). (B) Water contact angles of the studied composite hydrogels. Each point represents the mean ± SD (*n* = 3). P values were determined using an ordinary one‐way ANOVA with Dunnett's multiple comparison test, revealing statistically significant differences between MC/PEG and MC/PEG/PEGDA (*p* < 0.0001), MC/PEG and MC/PEG/PEGMA (p = 0.0007), and MC/PEG and MC/PEG/PAA (p = 0.0002). (C) Water contact angles evaluation. (D) Weight loss after immersion in PBS pH 7.4 of the hydrogels was analyzed for 60 days. Each point represents the mean ± SD (*n* = 3). (E) Compression stress–strain curves of the MC/PEG, MC/PEG/PEGDA, MC/PEG/PEGMA and MC/PEG/PAA hydrogels. (F) Average values of Young's modulus of the MC/PEG, MC/PEG/PEGDA, MC/PEG/PEGMA and MC/PEG/PAA hydrogels. Each point represents the mean ± SD (*n* = 5). P values were determined using an ordinary one‐way ANOVA with Dunnett's multiple comparison test, showing no statistically significant differences between MC/PEG and MC/PEG/PEGDA (p = 0.0780), MC/PEG and MC/PEG/PEGMA (p = 0.0758), and MC/PEG and MC/PEG/PAA (*p* > 0.9999). (G‐H) Representative images of the degradation of the hydrogels MC/PEG (G), and MC/PEG/PAA (H). An inert magnet was encapsulated in the polymer matrix as a surrogate model for simulating the incorporation of an implantable device. Samples were incubated in PBS buffer (pH 7.4) at 37°C to evaluate morphological and structural stability at 0, 10, 40, 50 and 60 days. Significance levels: *p* < 0.05 (^*^), *p* < 0.01 (**), *p* < 0.001 (^***^), *p* < 0.0001 (^****^); ns denotes not significant.

With the aim of comparing the different hydrogels in terms of their ability to control water uptake, swelling ratio (Figure 3A) and contact angle (Figure 3B,C) were estimated as parameters indicative of surface polarity and hydrophobicity. All systems showed an increase in contact angle compared to the MC/PEG hydrogel, suggesting a lower surface wettability. Specifically, the highest contact angle was observed on MC/PEG/PEGDA, possibly attributable to the PEGDA acrylic groups, determining higher surface compactness through the formation of non‐covalent interactions, such as hydrogen bonds, with the hydroxyl groups of MC and PEG. PAA could instead act by limiting the exposure of free polar groups. The analysis of the swelling capacity in water showed a clear inverse correlation with the contact angle: as hydrophobicity increases, the capacity to absorb water decreases. After the first 2‐hours exposure, the MC/PEG system, with the lowest contact angle value, showed the highest swelling (34%), while hydrogels containing PAA, PEGDA, and PEGMA showed a progressive reduction of the swelling ratio (between 10% and 25%), consistent with a higher surface density partly hindering the entry of water. The inherent tendency of the hydrogels to swell upon water absorption is also due to their porous structure, clearly revealed by SEM analysis (Figure S3).

Regarding the hydrogel capability to protect encapsulated devices from hydrolytic degradation, the evaluation of its stability under physiological conditions is a crucial aspect. It has been evaluated for a 2‐month period, monitoring the hydrogels weight loss every 10 days (Figure 3D; Figure S4). Coherently with swelling and wettability studies, MC/PEG was found to be the least stable system, with significant improvement to hydrolytic degradation provided by the added polymers. Nonetheless, although on all systems the degradation was particularly impactful after the first 20 days, on MC/PEG/PAA hydrogel, already starting from the seventh day, the degradation process seems to stabilize, indicating greater stability compared to the other formulations and highlighting the beneficial role of PAA in enhancing network cohesion and mitigating hydrolytic erosion under physiologically relevant conditions. The same was observed by visually monitoring the hydrogels degradation under simulated physiological conditions (Figure 3G,H; Figure S5). The entrapment of an inert object, namely a 1 cm Teflon magnet, was intended to mimic the encapsulated device. Similarly to weight loss evaluation, the MC/PEG/PAA revealed to almost fully preserve its structure after a 2‐months period, with the magnet inside still completely coated (Figure 3H), evidently providing higher stability under physiological conditions compared to MC/PEG alone (Figure 3G), which exhibits faster degradation kinetic leaving the object almost uncoated after 60 days.

The mechanical behavior of the hydrogels was evaluated by unconfined compression tests (Figure 3E,F). The Young's modulus (Figure 3F), calculated from the linear portion of the stress–strain curve (0–4% strain), ranged between 8 and 12 kPa for all formulations, consistent with the softness of native soft tissue, with no statistically significant differences observed (*p* > 0.05). The stress–strain curves (Figure 3E) showed that all samples could withstand large deformations, up to 80% strain, confirming their elastic nature. Specifically, MC/PEG and MC/PEG/PAA hydrogels reached the highest stress levels, close to 150 and 170 kPa, respectively, further confirming that the addition of PAA improves the mechanical properties of MC/PEG. Remarkably, the evidence of mechanical softness, tissue‐like elastic properties and high fracture toughness evidence the high suitability of MC/PEG/PAA in bioimplant applications, acting in reducing the mechanical mismatch at the biointerface and adapting to the dynamic deformations of the biological environment. Based on these results and of thermo‐responsivity, wettability, and stability evaluation, MC/PEG/PAA was selected as the hydrogel system to test as sensor protective coating, evaluating its antifouling properties and in vivo biocompatibility.

**FIGURE 4:**
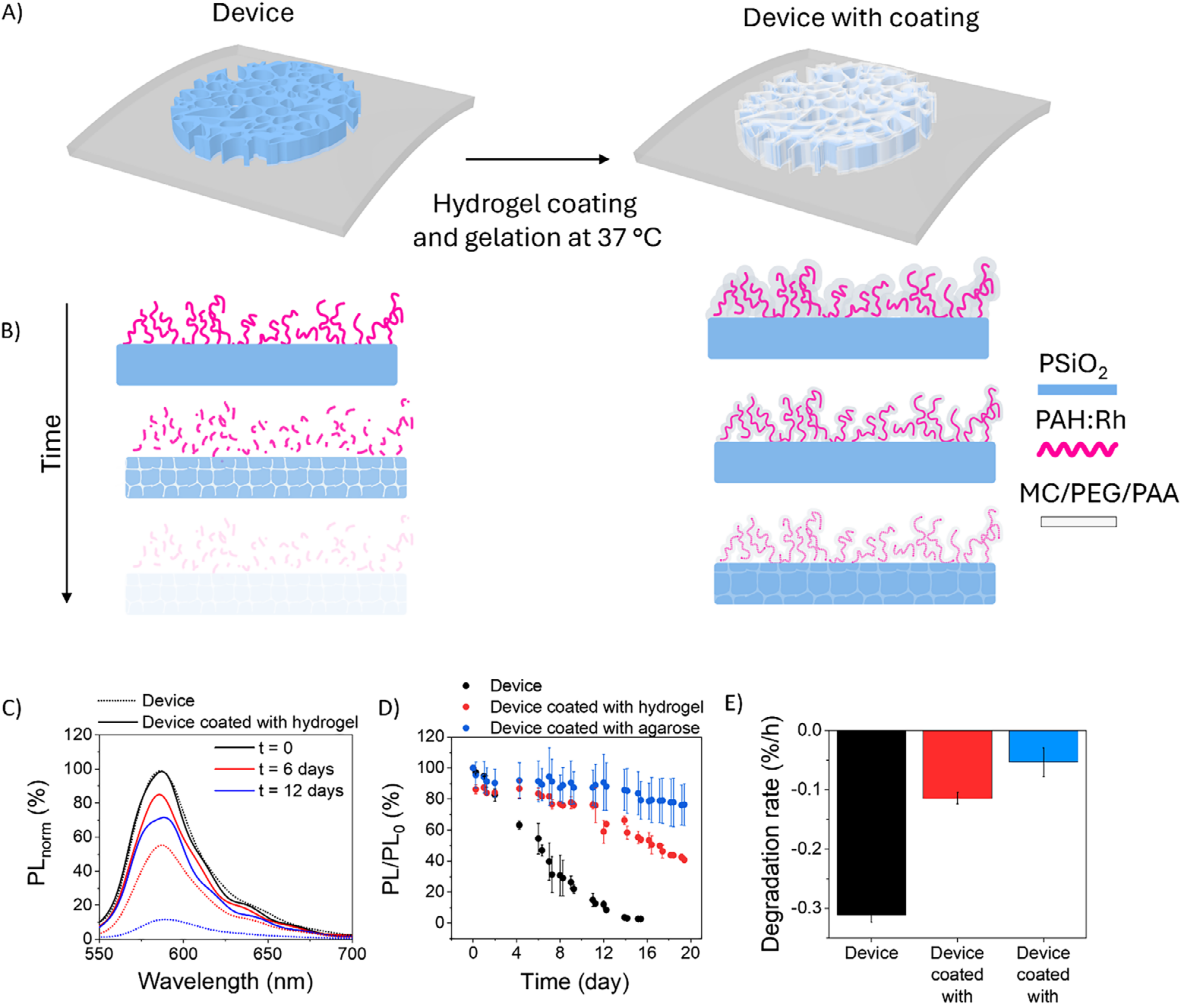
Evaluation of the MC/PEG/PAA hydrogel as a protective coating for porous silicon‐based optical device. (A) Schematic of the encapsulation system: the device consists of a nanostructured thermally oxidized porous silicon (PSiO_2_) substrate functionalized with PAH:Rh and coated with the hydrogel (MC/PEG/PAA). Upon incubation at 37°C, the hydrogel undergoes rapid gelation, forming a stable encapsulating layer. (B) Sketch of degradation mechanism for uncoated (left) and hydrogel‐coated (right) devices. The magnification shows the multilayer structure: PSiO_2_ sensor core (blue), PAH:Rh functional layer (pink), and the outer hydrogel coating (gray). (C) Representative photoluminescence (PL) spectra recorded in PBS at 37°C from uncoated device (dashed lines) and device coated with MC/PEG/PAA hydrogel (solid lines) at different monitoring time points, illustrating the temporal evolution of the emission signal. (D) Normalized PL intensity at 580 nm over time for devices without coating, and those coated with MC/PEG/PAA or agarose hydrogels. PL_0_ refers to the initial fluorescence signal immediately after encapsulation, used as reference. (E) Degradation rate of the different device configurations, calculated from the linear fitting of the curves in (D). Data in (D) and (E) are reported as mean values from three independent devices, with error bars representing standard deviation (SD).

To assess the functional performance of the thermo‐responsive MC/PEG/PAA hydrogel as a protective encapsulation layer, we fabricated a porous silicon‐based optical fluorescent device and embedded it within the hydrogel matrix. The device comprised a thermally oxidized porous silicon (PSiO_2_) scaffold functionalized with a nanometer‐thick fluorescent polyelectrolyte layer—poly(allylamine hydrochloride) labeled with Rhodamine B (PAH:Rh). Successful functionalization of the porous matrix was confirmed by interferometric reflectance spectroscopy, which revealed a stepwise increase in the effective optical thickness (EOT = 2n·t, where n is the effective refractive index and t the physical thickness) following PAH:Rh deposition, relative to the bare PSiO_2_ reference layer [54, 55] (Figure S6). Importantly, the sol state of the hydrogel at room temperature enabled facile encapsulation of the device via immersion coating, followed by a rapid sol‐to‐gel transition upon heating to 37°C (≈60 s). This process resulted in the formation of a stable, conformal hydrogel layer surrounding the fluorescent scaffold (Figure 4A,B). As a control, PAH:Rh‐functionalized PSiO_2_ scaffolds were also encapsulated with an agarose hydrogel layer that is non‐degradable under physiological conditions.

The hydrogel layer was designed to act as a protective barrier, limiting water penetration and preserving the structural and functional integrity of the fluorescent porous silicon (PSiO_2_) device relative to its uncoated counterpart (Figure 4B). In the absence of a protective layer, water infiltration promotes progressive hydrolithic dissolution of the PSiO_2_ scaffold, which destabilizes the surface‐bound PAH:Rh nanolayer and, consequently, diminishes its fluorescence emission over time. To assess the hydrogel protective performance, photoluminescence (PL) spectroscopy was carried out over a 20‐days period during which devices were immersed in phosphate‐buffered saline (PBS) at 37°C. Uncoated devices exhibited a rapid decline in PL intensity, with signal quenching nearly complete by day 16. In contrast, devices encapsulated with the MC/PEG/PAA hydrogel retained substantially higher fluorescence levels throughout the test period (Figure 4C,D). Normalized PL intensity at 580 nm (PL/PL_0_), where PL_0_ refers to the initial post‐encapsulation value, is shown in Figure 4D. While uncoated devices exhibited a steady and complete fluorescence loss, hydrogel‐coated devices preserved ≈60% of their initial signal after 16 days—comparable to control samples coated with agarose, a known barrier material that is non‐degradable under physiological conditions [56], which retained ≈80% of PL intensity over the same interval. To quantitatively compare protection efficacy, the degradation rate was estimated from the slope of the PL decay curves (Figure 4E). Agarose‐coated devices exhibited the lowest rate (≈0.05%/hour), followed by MC/PEG/PAA‐coated devices (≈0.1%/hour), which still represented a ≈3‐fold improvement over uncoated devices. These results confirm that the MC/PEG/PAA hydrogel effectively slows down PSiO_2_ degradation and extends device operational lifetime under physiological conditions.

This protective behavior originates from the transport characteristics of the MC/PEG/PAA network. Although water‐rich, such polyelectrolyte hydrogels can effectively limit water permeation through two complementary mechanisms. First, Donnan exclusion, arising from the fixed anionic groups of the PAA domains, which establish an electrostatic potential across the gel–solution interface. Under physiological ionic strength, this alters ion/water partitioning, reducing the osmotic pressure difference between the hydrogel and the surrounding medium, thus promoting an “osmotic deswelling” and a contraction of the network mesh [57, 58], which reduces transport to the device. Second, gel‐blocking, i.e., the formation of a highly swollen, low‐porosity interfacial layer that throttles further flux, produces a dense, swollen “skin” at the fluid entry face that closes interstitial pathways and lowers water permeability, as classically observed in PAA‐based absorbents [59, 60, 61, 62].

The selection of PAA as an additional polymer within MC/PEG matrix is further supported by its well‐known biocompatibility [63, 64, 65]. Additionally, the negatively charged PAA domains can act in modulating surface charge, reducing non‐specific protein adsorption, thereby improving the overall cytocompatibility of the matrix. Also, the biocompatible PEG is expected to improve the interfacial characteristics of the hydrogel by reducing non‐specific cell adhesion through the formation of a hydrated, protein‐repellent layer [65, 66, 67, 68].

**FIGURE 5:**
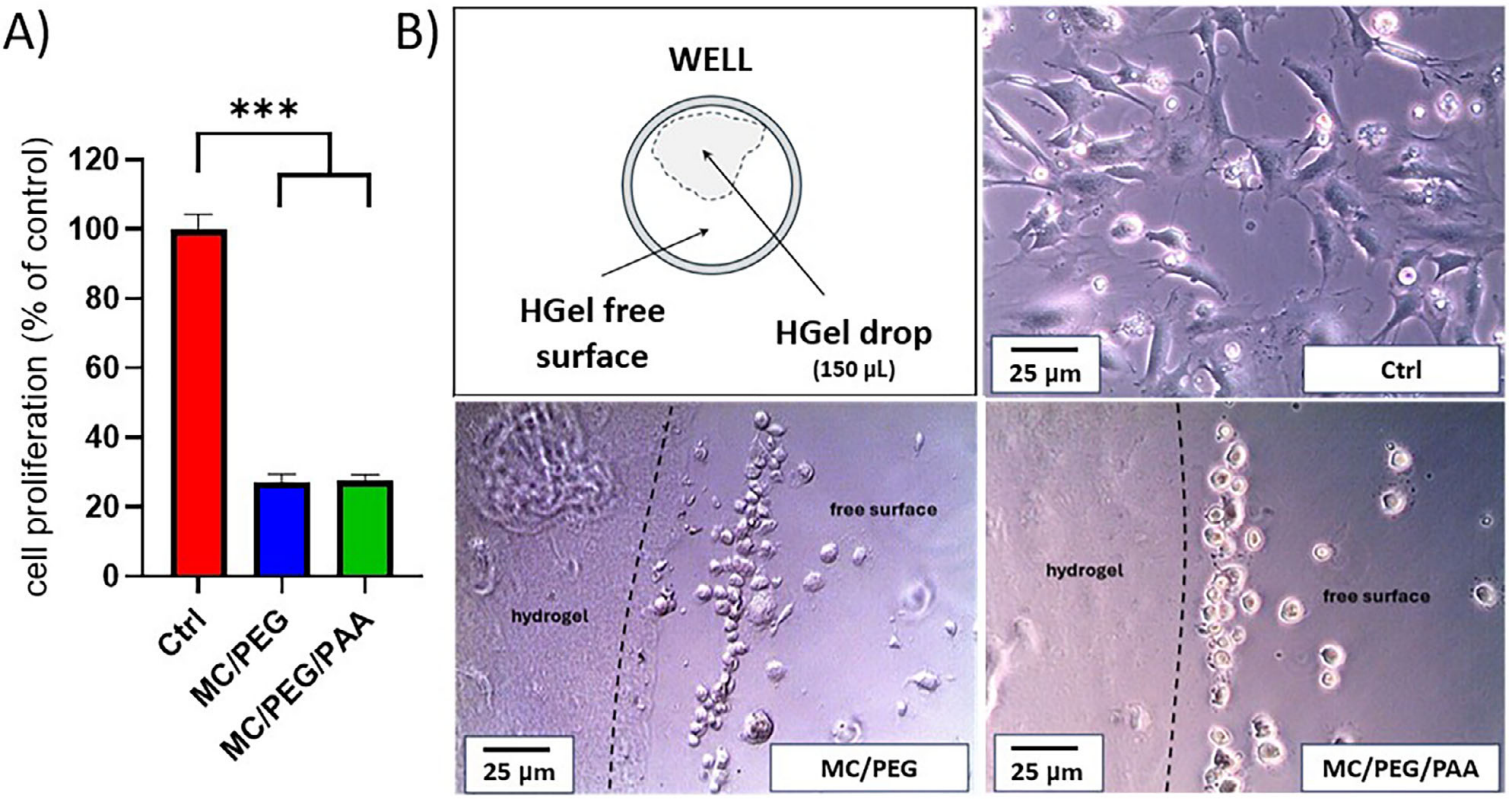
Analysis of proliferation and adhesion of NIH/3T3 mouse fibroblasts on the surface of MC/PEG and MC/PEG/PAA hydrogels. (A) Analysis of cell metabolic activity/proliferation by MTT assays assessed in NIH/3T3 cells grown in the presence or absence of hydrogel, for 24 h. Data are represented as percent values with respect to the untreated control cells grown in the absence of hydrogel (Ctrl = 100%). Data are reported as mean values ± S.E.M; statistical analysis: two‐tailed unpaired Student's *t*‐test (*n* = 9; ^***^ *p* < 0.001). (B) Representative pictures of NIH/3T3 fibroblasts grown in the absence (Ctrl cells, no hydrogel (HGel)) or in the presence of MC/PEG and MC/PEG/PAA hydrogels. In pictures, the dashed black lines run along the boundary between the hydrogel‐covered area and the hydrogel‐free surface. Magnification: 40X; scale bar: 25 µm. In the top left box: drawing of the hydrogel droplet deposition inside the well before cell seeding.

This was confirmed by cell‐based assays, specifically performed to evaluate the MC/PEG/PAA hydrogel ability to prevent fibroblast adhesion, thus limiting pro‐fibrotic and/or inflammatory response of the body. Proliferation and adhesion of mammalian fibroblasts, with respect to MC/PEG, were assessed by MTT assays, by using the mouse‐derived NIH/3T3 fibroblast cell line. As represented in Figure 5A, after 24 h of cell culture, the presence of MC/PEG/PAA hydrogel significantly inhibits fibroblast metabolic activity and proliferation with respect to control cells grown in the absence of hydrogel (27.7 ± 1.6% vs. 100 ± 4.3%, respectively). Moreover, similar metabolic activity and proliferation data have been found for MC/PEG/PAA compared with MC/PEG hydrogel (27.3 ± 2.1% vs. 100 ± 4.3% of control), thus suggesting the neutrality of the PAA structural modification in terms of cellular behavior. In addition to quantitative data on proliferation, a qualitative assessment of cell adhesion was conducted. As shown in Figure 5B, in the presence of both MC/PEG and MC/PEG/PAA hydrogel drops deposited inside the growth area, murine fibroblasts were unable to adhere to the hydrogel surface, remaining crowded and aligned along the border between the hydrogel and the free growth surface in the well. Furthermore, it is possible to note that cell adhesion dynamics are inhibited and/or delayed even on the side of the growth surface immediately adjacent to the hydrogel, contrarily to control fibroblast cells showing a regular morphological phenotype. This evidence is in line with cell proliferation analysis, given that cell adhesion is a key premise for regulating the subsequent cell proliferation process as well as the proliferation/differentiation switching program [69]. Overall, the in vitro data support the hypothesis that the investigated MC/PEG/PAA hydrogels, since being non‐permissive toward the adhesion of fibroblast‐like cells, can act in avoiding the onset of fibrotic formations and processes [70].

**FIGURE 6:**
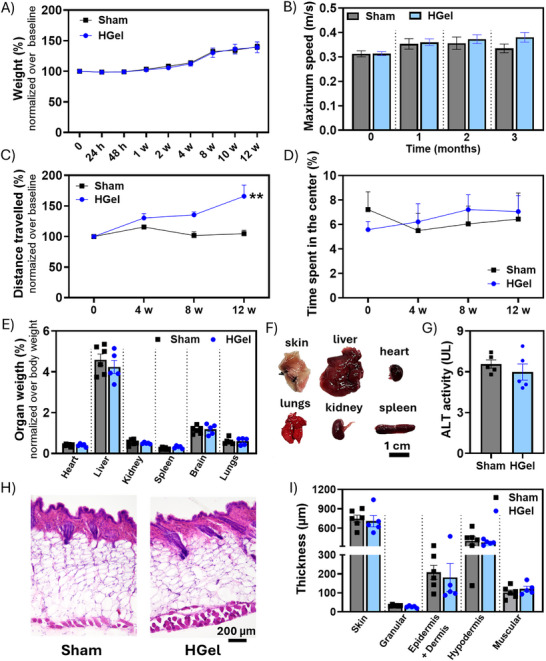
In vivo and ex vivo biocompatibility assays. (A) Weight gain expressed over body weight at baseline (%) for Sham (*n* = 6) and mice implanted with MC/PEG/PAA hydrogel (*n* = 5) from 24 h to the 12th week after implantation (Two way repeated measure ANOVA, sex as covariate; Type of implant p = 0.789; Sex p = 0.719; Time *p* < 0.001; Time*Type of implant p = 1.0). (B) Maximum speed, (C) total distance travelled normalized over baseline (%) and (D) % of time spent in the center in OF test at baseline and 4, 8 and 12 weeks after implantation in Sham (*n* = 6), Hydrogel (*n* = 5)‐implanted mouse (Two way repeated measure ANOVA, sex as covariate; Maximum speed: Type of implant p = 0.146; Sex p = 0.046; Time p = 0.565; Time* Type of implant p = 0.028; Distance travelled: Type of implant p = 0.170; Sex p = 0.236; Time p = 0.311; Time* Type of implant p = 0.007; Percentage of time spent in the center of arena: Type of implant p = 0.564; Sex p = 0.171; Time p = 0.421; Time* Type of implant p = 0.728). (E) Organ weight analysis at the end of the observational period revealed no significant differences between the two groups, indicating a higher biocompatibility index. For each animal, organ weight was normalized over body weight (%). Sham, *n* = 6; Type of implant, *n* = 5 (Two‐way repeated measure ANOVA, sex as covariate; Type of implant p = 0.921; Sex p = 0.145; Organ type p < 0.001; Organ type* Type of implant p = 0.995). (F) Images of harvested organs after 3 months from implantation: skin, liver, heart, lungs, kidney and spleen. (G) Analysis of serum alanine aminotransferase (ALT) activity (U/L) as an index of hepatic function in Sham (*n* = 5) and Hydrogel (n = 5) mouse groups. (Univariate test, sex as covariate; Type of implant p = 0.701; Sex p = 0.35). (H) Representative H&E‐stained sections of skin from sham (*n* = 6) and hydrogel‐implanted mice (*n* = 5). (I) Total skin thickness and thickness of individual skin layers (granular, epidermis + dermis; hypodermis; and muscular) from histological images of sham mice (*n* = 6) and hydrogel (*n* = 5). (Total skin thickness: Univariate ANOVA; Type of implant p = 0.707; Thickness (µm) of individual skin layers: Two‐way repeated measure ANOVA; Type of implant p = 0.700; skin layers *p* < 0.001; skin layers* type of implant p = 0.938). All data represented as mean ± S.E.M.; considering *p* < 0.05 as significant.

In vivo safety and biocompatibility of the MC/PEG/PAA hydrogel were evaluated with a comprehensive study conducted over a 12‐week period in mice implanted with the hydrogel and sham control mice. Body weight was monitored weekly throughout the study. As shown in Figure 6A, no statistically significant differences in weight variation were observed between hydrogel‐implanted and control animals at any timepoint, aside from the expected physiological weight gain (see Table S2 for statistical analysis). According to FELASA guidelines, a body weight loss exceeding 20% indicates severe discomfort; in our study, fluctuations remained well within the physiological range, indicating the absence of systemic distress.

To assess potential behavioral alterations, open field (OF) tests were conducted pre‐surgery (baseline, 0) and at 4, 8, and 12 weeks post‐implantation. Analysis of total distance travelled revealed a significant time‐by‐group interaction (Figure 6C), yet no significant differences were observed in the maximum speed (Figure 6B) and percentage of time spent in the centre of the arena (Figure 6D), suggesting that neither locomotor activity nor anxiety‐like behavior was adversely affected by the implanted hydrogel.

General health status was further evaluated through the SHIRPA protocol, which included observations of activity level, tremors, lacrimation, eyelid closure, fur condition, whisker movement, and defecation. No deviations from normal behavior were noted throughout the entire observation period (data not shown), supporting the absence of overt systemic toxicity. By visually monitoring the animals during the implantation period, no evidence of hydrogel leaching from the implantation site was observed. Additionally, macroscopic observation of the tissues above the implantation site revealed no alterations.

At the end of the 12‐week‐long period, animals were euthanized, and major organs—including liver, spleen, kidneys, lungs, heart, brain, and the skin at the implantation site—were harvested for macroscopic evaluation and weight analysis. Interestingly, no residues of hydrogel could be visually detected, suggesting its complete bioresorption. No significant differences in organ weights were detected between groups (Figure 6E), and no gross morphological abnormalities were observed (Figure 6F), further corroborating the biocompatibility of the hydrogel. Serum alanine transaminase (ALT) levels, a key biomarker of liver function and systemic toxicity, were measured at sacrifice. No significant differences in ALT activity were observed between sham and hydrogel groups (Figure 6G), indicating preserved hepatic function.

Transverse cryosections of skin (40 µm thick) were stained with hematoxylin and eosin (H&E). As shown in Figure 6H, histological analysis revealed no significant alterations in the morphology of the entire tissue and in overall skin thickness or in the organization of its layers when compared with sham mice, thereby excluding local tissue toxicity (Figure 6I). Moreover, no hydrogel residues were detected, confirming complete bioresorption after 3 months. A representative histological image with annotated skin layers is provided in the Supporting Information as a reference (Figure S7). These findings confirm the excellent biocompatibility, non‐toxicity, and complete bioresorbability of the MC/PEG/PAA hydrogel in vivo.

**FIGURE 7:**
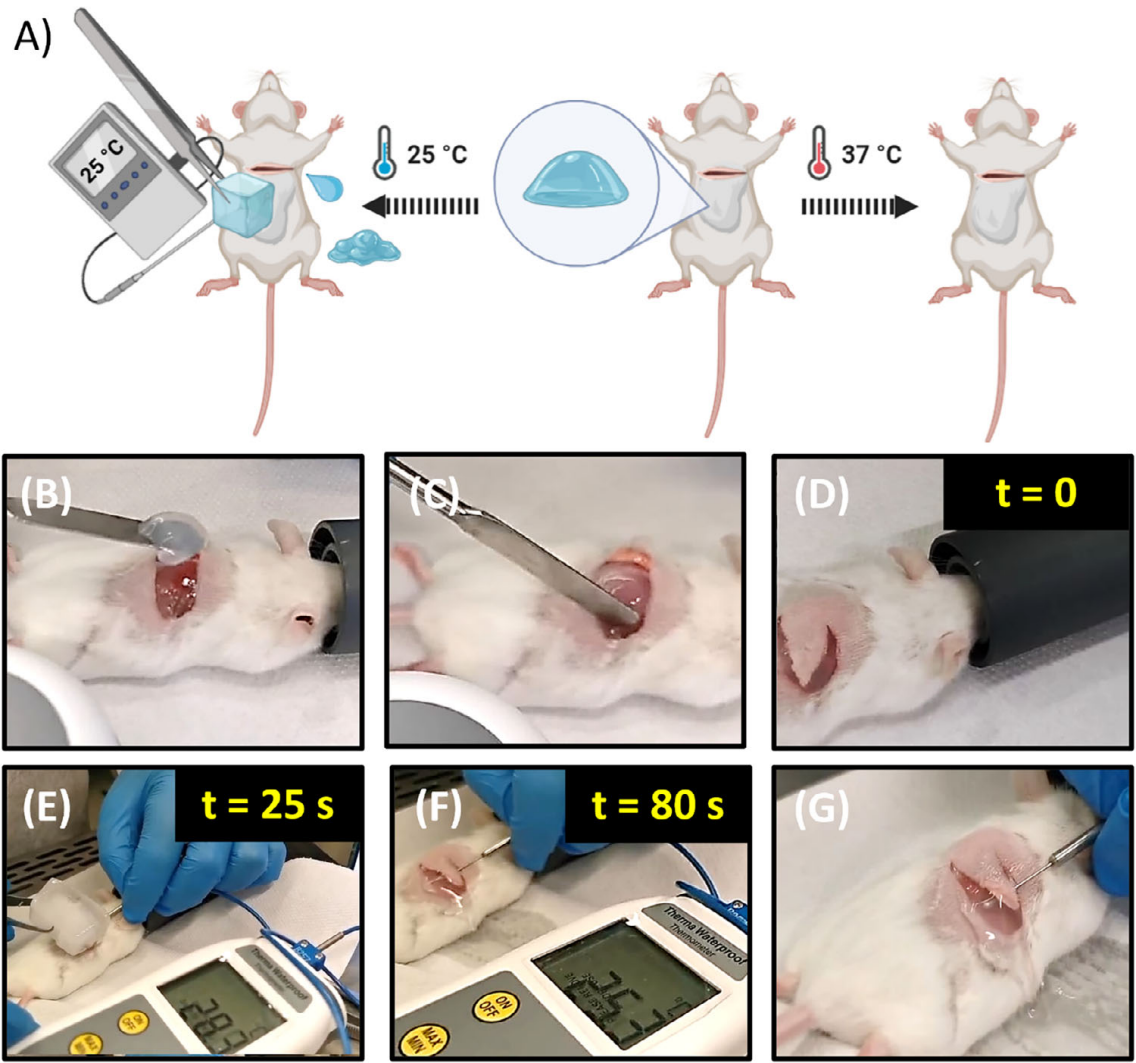
In vivo evaluation of on‐demand thermal degradation of MC/PEG/PAA hydrogel. (A) Schematic representation of the in vivo evaluation of MC/PEG/PAA hydrogel on‐demand thermal degradation. The hydrogel was manually placed into a surgically prepared subcutaneous pocket. Localized external cooling (≈25°C) was then applied to induce gel–sol transition and complete dissolution of the material (left). Control animals received no thermal stimulus (right). (B–D) The hydrogel is implanted in the back of mice without suturing the pocket to visually check the gel‐sol transition. (E,F) An ice cube is applied in contact with the skin of the pocket (T ≈25°C). The time‐lapse at different time intervals shows the thermoresponsive behavior of the MC/PEG/PAA implanted hydrogel. A localized temperature decrease to approximately 25°C induces a visible gel‐to‐sol transition with complete hydrogel dissolution. A magnification of the subcutaneous area with the dissolved hydrogel is shown in (G).

The key feature of the hydrogel, consisting of on‐demand temperature‐triggered degradation, was verified in vivo, after its implantation in a surgically prepared subcutaneous pocket, as shown in Figure 7A–D. Upon localized cooling to approximately 25°C, the hydrogel underwent rapid gel‐sol transition, resulting in complete dissolution in less than 2 min (Figure 7E–G), highlighting the high responsivity of the system to slight thermal variations. In contrast, in control animals maintained at physiological temperature (Figure S8), the implanted hydrogel preserved its structural integrity throughout the observation period, indicating high in vivo stability in the absence of thermal input. Overall, these results suggest that this hydrogel system allows for on‐demand removal via a non‐invasive external spatially confined thermal input.

## 3 Statistical Analysis

### 3.1 Statistical Analysis of Data (Hydrogel Characterization Studies)

Statistical analyses were performed using GraphPad Prism software (version 10.4.1). Data are presented as mean ± standard deviation (SD), unless otherwise stated. Weight loss measurements over time (*n* = 3) were analyzed using two‐way ANOVA with Dunnett's multiple comparison test. Water contact angles (*n* = 3) and average Young's modulus values (*n* = 5) were analyzed using ordinary one‐way ANOVA with Dunnett's multiple comparison test. Each data point represents an independent sample. Statistical significance thresholds were set at p < 0.05, with significance levels indicated as follows: *p* < 0.05 (^*^), *p* < 0.01 (^**^), *p* < 0.001 (^***^), *p* < 0.0001 (****); ns denotes not significant (*p* > 0.05).

### 3.2 Statistical Analysis of Data (In Vivo Studies)

Data are presented as mean ± standard error of the mean (S.E.M.). Group differences were analyzed using univariate ANOVA or repeated measures ANOVA (with type of implant and time as between and within factors, respectively), as appropriate. Sex was used as a covariate. Statistical analyses were conducted using SPSS software (version 26) with *p* < 0.05 considered significant (*p* < 0.05 ^*^; *p* < 0.01 ^**^; *p* < 0.001 ^***^) and 0.10 > *p* > 0.05 indicating a trend toward significance (#).

### 3.3 Animal Authorization

All procedures were approved by the Animal Health and Care Committee of the University of Modena and Reggio Emilia, in accordance with National Institutes of Health guidelines [CEE Council 89/609; Italian DL 26/2014, authorization no. 979/2020/PR]. Efforts were made to minimize animal suffering and the number of animals used.

## 4 Conclusion

Here we present a thermoresponsive, bioresorbable hydrogel based on methylcellulose, polyethylene glycol, and polyacrylic acid (MC/PEG/PAA), developed as a transient coating for implantable sensors. This physically crosslinked system avoids cytotoxic reagents and complex synthesis, enabling a reversible gel–sol transition triggered by a mild thermal stimulus, consisting in temperature decrease to about 25°C, while remaining stable at physiological temperature. Hydrogel ability in preserving signal transmission is demonstrated through the encapsulation of a model fluorescent device, showing enhanced signal stability in comparison to the uncoated one. Antifibrotic properties and high biocompatibility were verified by cell‐based and in vivo assays, respectively, also demonstrating the effective on‐demand transition in vivo, triggered by a slight temperature decrease. The developed coating offers a safe, controllable, and clinically relevant strategy for temporary sensor encapsulation, overcoming critical limitations of materials currently used in implantable technologies and contributing to revolutionize patient‐centred healthcare.

## Author Contributions

E.M. and F.P. contributed to the conceptualization of the study. Methodology and investigation were carried out by F.P., E.V., E.D., A.V., M.C., L.L., M.F., L.P., and A.B. Visualization was performed by E.M., D.G., G.B., C.D., F.P., T.V., and C.M. Supervision and funding acquisition were provided by E.M. The original draft of the manuscript was written by F.P., E.V., E.D., A.V., M.C., L.L., M.F., L.P., and A.B., while review and editing were conducted by E.M., D.G., G.B., C.D., F.P., C.M., and T.V.

## Conflicts of Interest

The authors declare no conflict of interest.

## Supporting information




**Supporting File**: advs73655‐sup‐0001‐SuppMat.docx.

## Data Availability

The data that support the findings of this study are available from the corresponding author upon reasonable request.
