# Supplementary material for: Thermally Degradable Biocompatible Hydrogel as Transient Encapsulation Coating for Implantable Sensors
Source: Adv Sci (Weinh). 2026 Apr 16;13(30):e17202. doi: 10.1002/advs.202517202 (PMC13248840; doi:10.1002/advs.202517202)
Supplement: Supplementary file 1 — Supporting File: advs73655‐sup‐0001‐SuppMat.docx. [file ADVS-13-e17202-s001.docx]

**Supporting Information**

**Thermally Degradable Biocompatible Hydrogel as Transient Encapsulation Coating for Implantable Sensors**

Francesca Persano^1^, Eleonora Vandini^2^, Eleonora Daini^2^, Antonietta Vilella^2^, Daniela Giuliani^2^, Leonardo Lamanna^3^, Marco Friuli^3^, Christian Demitri^4^, Laura Pecoraro^5^, Amilcare Barca^6^, Tiziano Verri^5^, Cosimino Malitesta^1^, Martina Corsi^7^, Giuseppe Barillaro^7^, Elisabetta Mazzotta^1,*^

^1^ *Laboratory of Analytical Chemistry, Department of Biological and Environmental Sciences and Technologies (Di.S.Te.B.A.), University of Salento, Lecce (Italy)*

^2^ *Department of Biomedical, Metabolic and Neural Sciences, University of Modena and Reggio Emilia, Via G. Campi 287, Modena, 41125 Italy*

^3^ *Department of Engineering for Innovation, Campus Ecotekne, University of Salento, Lecce (Italy)*

^4^ *Department of Experimental Medicine, University of Salento, Lecce (Italy)*

^5^ *Laboratory of Applied Physiology, Department of Biological and Environmental Sciences and Technologies (Di.S.Te.B.A.), University of Salento, Lecce (Italy)*

^6^ *Laboratory of Applied Physiology, Department of Experimental Medicine (Di.Me.S.), University of Salento, Lecce (Italy)*

*^7^ Department of Information Engineering, University of Pisa, via G. Caruso 16, 56122 Pisa, Italy.*

Corresponding Author: [elisabetta.mazzotta@unisalento.it](mailto:elisabetta.mazzotta@unisalento.it)

**Experimental sections**

**Materials and chemicals**

Methyl cellulose (MC, viscosity 4,000 cP) was purchased from Sigma Aldrich. Sodium chloride (NaCl, ≥ 99.0%); poly(ethylene glycol) (PEG, average Mn 4,600); poly(ethylene glycol) diacrylate (PEGDA, average Mn 575); poly(ethylene glycol) methyl ether acrylate (PEGMA, Mn 480); poly(acrylic acid) (PAA, viscosity ≤ 2,000 cP) were purchased from Sigma Aldrich.

Boron-doped silicon wafers (p-type, single-side polished, orientation <100>, resistivity 0.8–1.2 mΩ cm) were used as substrates for sensor fabrication (Siltronix Silicon Technologies, France). Aqueous hydrofluoric acid (HF, 48%), sodium hydroxide (NaOH, 98%), aqueous hydrochloric acid (HCl, 37%), sodium acetate (CH_3_COONa, 99%), sodium phosphate monobasic monohydrate (PBS, 98%), tris(hydroxymethyl)aminomethane (TRIS, 99%), sodium chloride (NaCl, 99%), sodium phosphate, (Na_2_HPO_4_, 99%), Agarose were purchased from Merck-Sigma Aldrich (Germany). Poly(allylamine hydrochloride) labelled with Rhodamine (PAH:Rh, LD 1:126) was purchased from Surflay Nanotech (Germany). Absolute ethanol (EtOH, 99.9%), isopropyl alcohol (IPA, 99.5%), and diethyl ether (Et2O, > 99%) were purchased from Carlo Erba Reagents. Aqueous solutions were prepared in deionized water (DIW, 15 MΩ×cm) filtered by Elix® (Merck Millipore). All buffers were prepared in DIW, and their pH adjusted with NaOH (5 M) and HCl (5.4 M) aqueous solution.

**Preparation of MC/PEG-based hydrogels**

MC solution at a concentration of 1.5% (w/v) was prepared by dispersing MC powder in hot water at 70 °C under continuous magnetic stirring until a homogeneous dispersion was obtained. The resulting solution was then stored at 4 °C for 48 h to ensure a clear and transparent solution. NaCl at a concentration of 6% (w/v) and PEG at 10% (w/v) were incorporated into the MC solution to prepare the in situ gelling solution. The resulting hydrogel was named MC/PEG.

To prepare MC/PEG/PEGDA and MC/PEG/PEGMA hydrogels, 10% (v/v) PEGDA and PEGMA were added to the 1.5% (w/v) MC solution, respectively, resulting in solutions with a final MC concentration of 1.35% (w/v). The required amount of PEG and NaCl was added to the MC/PEGDA and MC/PEGMA solutions. A PEG concentration of 10% (w/v) and a NaCl concentration of 6% (w/v) were used for this step.

Finally, to prepare the MC/PEG/PAA hydrogel, a MC solution (at a concentration of 1.5% (w/v) was first prepared as described above. Then, the required amount of PEG, NaCl, and PAA was added to the MC solution. A PEG concentration of 10% (w/v), a NaCl concentration of 6% (w/v), and a PAA concentration of 1% (w/v) were used.

**Inversion tube test**

To assess the thermosensitive sol-gel transition using the inversion tube test, the solutions were placed in glass vials at varying temperatures. The fluidity/viscosity of the hydrogel was evaluated through visual inspection using the tilt method at different experimental time points.

**Rheological characterization**

Rheological measurements were conducted using a Malvern Kinexus rheometer, equipped with a parallel plate geometry (plate diameter 25 mm, gap distance fixed at 0.25 mm). Immediately after preparing the hydrogel samples, a temperature ramp was applied to assess the changes in the storage modulus (G′) and loss modulus (G″) for the different samples, measured between 5 and 50 °C. These measurements were carried out with a shear strain of 3% and a frequency of 1 Hz, with the temperature increased at a rate of 1 °C/min. The gel point was determined by the intersection of the G′ and G″ curves ^[1,2]^. To evaluate the hydrogels' thermo-reversibility, the samples were heated to 37 °C for the duration required for gelation. All measurements were performed starting at 40 °C, then cooling to 5 °C or 2.5 °C at a rate of 1 °C/min. Lastly, to determine the time for the sol-gel transition, the hydrogel solutions were kept isothermal at 37 °C, maintaining all other parameters constant.

The ability of the MC/PEG/PAA formulation to maintain structural integrity under increasing stress in physiological conditions was evaluated with a strain amplitude sweep test performed at 37 °C and 1 Hz, applying a strain ranging from 0.1% to 100%.

**Contact angle measurements**

The hydrophobicity of the optimized hydrogels was determined by First Ten Angstroms, FTA 1000 software (Newark, California, USA) equipped with a CDD camera using the sessile drop method at room temperature. The static contact angle between the water droplet and the surface of the hydrogel was measured. Three measurements for each formulation were done and an average value was calculated.

**Swelling studies**

The swelling behavior of the hydrogels was evaluated through gravimetric analysis. In brief, after gelation at 37 °C, the initial weight of the samples was recorded using an analytical balance. The samples were then submerged in PBS at 37 °C, and their weight was measured at various time intervals. The percentage of swelling ratio (SR) was calculated according to the following formula:

SR (%) = [(W_wet_ - W_dry_)/W_dry_] x 100……………………………………(1)

W_dry_ is the hydrogel's initial dry weight and W_wet_ is the weight of the hydrogel after hydration in PBS at 37 °C. The readings were performed in technical triplicates.

**Degradation studies in simulated physiological conditions**

The hydrolytic degradation of the four time-optimized formulations was evaluated under simulated physiological conditions through stability testing. The fabricated hydrogels were incubated in PBS for 24 hours to reach swelling equilibrium. The samples were then hydrated in PBS at pH 7.4 and incubated at 37 °C, with weight measurements taken at different intervals. The percentage of weight loss (WL) was calculated using the following formula:

WL (%) = [(W_0_ - W_t_)/W_0_] x 100……………………………………… (2)

where W_0_ is the initial weight of the hydrogel at t = 0 after gelling and immersion for 24 h in PBS and W_t_ is the weight of the hydrogel at different time points. Degradation tests were performed in technical triplicates.

**Compression test**

To assess the stiffness of the hydrogel, the samples were subjected to an unconfined compression test. Briefly, the samples were tested after gelling at 37 °C; the initial diameter and thickness were recorded, and the sample was then placed between two waterproof, non-lubricated compression plates. Compression testing was performed at room temperature using a universal uniaxial testing machine (Zwick Roell, Ulm, Germany), equipped with a 10 N load cell, up to 80% strain, with a preload of 0.01 N and a displacement rate of 1 mm/s. The average Young's modulus (E) was determined as the slope of the linear portion of the stress-strain curves at low strain values (0–4%) for each hydrogel formulation.

**Morphological analysis**

For the morphological characterization of the four formulations, the hydrogels were frozen in liquid nitrogen and dried with a freeze drier (LIO 5P, Cinquepascal, Milan, Italy), and observed through a scanning electron microscope SEM at different magnifications (Zeiss, Sigma VP, Carl Zeiss, Jena, Germany).

**Assembly of PSiO₂ substrates coated with PAH:Rh**

***Fabrication, Oxidation of Porous Silicon Substrates and coating with PAH:Rh***

Porous silicon (PSi) substrates were fabricated at room temperature (RT) using a two-step electrochemical etching process in a 3:1 v/v HF:EtOH aqueous solution. A custom-designed Teflon cell equipped with a platinum wire cathode and a flat aluminum anode was used to etch a circular area of 0.567 cm² on the silicon wafer. The etching current was applied using a source measurement unit (SMU, Keithley 2602A). A first PSi sacrificial layer was carried out at a constant current density of 350 mA/cm² for 20 seconds and was rinsed in ethanol for 120 seconds, followed by immersion in a 1 M NaOH:EtOH (9:1 v/v) solution for 120 seconds to promote nanostructure formation and prevent formation of a parasitic layer that restrict the diffusion inside the PSi layer underneath. A final rinse in DIW and ethanol was performed to eliminate residues. For the second etching step, the duration was increased to 40 seconds and was rinsed sequentially in ethanol and diethyl ether (120 seconds each) to ensure a crack-free structure. Finally, the porous silicon membranes were oxidized in a muffle furnace (ZB/1, ASAL) at 1000 °C for 5 minutes in room atmosphere to convert the PSi into thermally oxidized porous silicon (PSiO₂).

PSiO_2_ substrates were coated with 50 ml of PAH:Rh solution (1 mg/ml in TRIS buffer 50 mM and 200 mM NaCl at pH 8), rinsed with DIW for 1 minute and dried with nitrogen flow.

***Reflectance Spectroscopy***

Reflectance spectra of the PSi and PSiO₂ substrates were collected in the 400–1000 nm range through the fiber probe coupled to an ultraviolet-visible (UV-VIS) spectrometer (USB2000+ UV-VIS, Ocean Optics) using deuterium and tungsten halogen lamps (DH-2000-BAL, OceanOptics) as light source. Spectra were recorded in reflection mode with an integration time of 2 ms and a spectral resolution of 0.35 nm. The spectral profile of the light source was corrected by normalizing to the reflectance spectrum of a silver mirror (PF10-03P01-Ø1, Thorlabs, USA). Contributions from ambient light were subtracted. Porosity, thickness, and effective optical thickness (EOT) of the PSi and PSiO_2_ substrates were extracted by fitting the reflectance spectra using a Fabry-Pérot interferometric model to the reflectance data using home-made software routines ^[3]^.

***Photoluminescence Spectroscopy***

Photoluminescence spectra (PL) were collected from wet PSiO₂ substrates coated with PAH:Rh. Emission was measured in the 400–1000 nm range using a green laser diode (CP520, λₑₓ = 520 nm, Thorlabs, USA) as the excitation source. The laser beam was directed with an incident angle of ~30° to the surface through a bifurcated fiber optic probe (QR200-7-SR, Ocean Optics, USA). Emitted light was collected via the second arm of the fiber and directed to a UV-VIS spectrometer (USB2000+UV-VIS, Ocean Optics, USA) using an optical fiber (M35L01, Thorlabs, USA). Spectra were recorded in scope mode with the following parameters: integration time 300 ms, 3 scans averaged, and boxcar width set to 1.

**Application and Evaluation of MC/PEG/PAA Hydrogel as Protective Coating**

***Preparation of Agarose Hydrogel***

Agarose was used as an ideal encapsulation material due to its hydrogel properties and low degradation rate. A 2% w/v agarose solution was prepared by dissolving 1 g of agarose powder in 50 mL of DIW. The solution was heated above the agarose melting point and vigorously stirred at 500 rpm for 2 hours until complete dissolution. Once fully dissolved, the hydrogel solution was ready for application. The agarose hydrogel was cooled to RT and stored at 4 °C until use. The agarose hydrogel was gelled at 37 °C for 30 minutes.

***Hydrogel Coating of the Sensor***

The PSiO₂ substrate coated with PAH:Rh was mounted onto a plastic Petri dish lid and secured using a PDMS O-ring (prepared with a 10:1 w/w base-to-curing-agent ratio, cured at 90 °C for 1 hour) with an external diameter of 6 cm, internal diameter of 1.2 cm 3 mm thick. This hole cavity was filled with the hydrogel in liquid form and gelled as reported in paragraph “*Preparation of Agarose Hydrogel*” and “*Preparation of MC/PEG-based hydrogels”* (see *MC/PEG/PAA Hydrogel*).

The hydrogel’s ability to act as an encapsulating layer was evaluated over time by monitoring the photoluminescence signal of the sensor (as reported in the section “*Photoluminescence Spectroscopy*”), which served as an indicator of sensor integrity and degradation protection.

Control sensor with and without hydrogels were immersed in 40 mL of phosphate-buffered saline (PBS, pH 7.4) maintained at 37 °C. Measurements were conducted twice daily and the PL signals from three independent sensors were measured at each time point with buffer replacement after each measurement session.

**Cell Culture and Treatments on NIH/3T3 mouse fibroblasts**

Mouse-derived NIH/3T3 fibroblast cells (ATCC CRL-1658) were used to evaluate metabolic activity and proliferation of cells growing in adhesion in the presence of the hydrogels. Cells were maintained in a humidified atmosphere (5% CO_2_ in air), at 37◦C, in DMEM medium supplemented with 10% (v/v) fetal bovine serum (FBS), 2 mM L-glutamine and 100 μg/mL penicillin-streptomycin solution. Cells were detached and harvested with a 0.3% (v/v) trypsin solution and then transferred to new flasks every 2–3 days (70–90% confluence) for propagation. The culture medium was changed every 2 days. All experiments were performed between passages 3 and 10 of propagation.

The MTT assay (3-[4,5-dimethylthiazol-2-yl]-2,5-diphenyl tetrazolium bromide, Sigma-Aldrich) was used to evaluate metabolic activity/proliferation of mouse fibroblasts in the presence or absence of the hydrogel. First, 150 μL hydrogel volume per well was deposited in 12-well plates. Then, the plates were sterilized by exposure to UV light for 30 min. 1 mL of cell culture medium was added to each well and the plate was incubated for 24 h at 37◦C to allow release of any soluble factor. Cells were seeded at a density of 2.0 × 10^5^ cells/mL in each well (0.5 mL per well) and incubated at 37◦C. At 24 h, MTT solution (5 mg/mL in sterile filtered PBS, pH 7.4) was added to each well to reach a final concentration of 0.5 mg MTT/mL, and plates were incubated at 37◦C for 3 h. The dark-blue formazan crystals were then solubilized by cell lysis with 700 μL/well 2-propanol/HCl 4N, and absorbance was measured at 550 nm with a Multiskan Fc Microplate Photometer (Thermo Fisher Scientific, Waltham, MA, USA). Data were reported as a percentage of control (mean ± SEM, n = 9 independent biological replicates).

**In vivo experiments**

***Animals***

Three-month-old (at the start of the experiment) CD1 (n=11) male and female mice were used for this study. The animals were purchased from the colony of Charles River Laboratories Italia s.r.l. (Calco-Milano, Italy) and housed in a pathogen-free facility with stable conditions of temperature (21±0.5°C) and humidity (60%). They were maintained on a 12-hour light/dark cycle with food and water available *ad libitum*. All procedures were approved by the Animal Health and Care Committee of the University of Modena and Reggio Emilia, in accordance with National Institutes of Health guidelines [CEE Council 89/609; Italian DL 26/2014, authorization no. 979/2020/PR]. Efforts were made to minimize animal suffering and the number of animals used.

***Hydrogel implantation procedure***

Animals were divided into two experimental groups, maintaining gender proportions: the Sham group (mice that underwent surgery without material implantation) and the hydrogel group (mice subjected to hydrogel implantation). To be implanted, the hydrogel was incubated for 48 hours at 37°C and then transferred to the surgical room on a heated plate.

During surgical procedures, mice were anesthetized with inhaled isoflurane and shaved on the back prior to surgery. The upper back skin was disinfected with Betadine, lifted, and incised to create a subcutaneous pouch of 1.5 × 1.5 cm for hydrogel sample (1 g) insertion. The incision was sutured using surgical stitches and treated with Neuflan gel (Neomycin 0.5 g/Fluocinolone acetonide 0.025 g/Lidocaine 2.5 g) to minimize postoperative pain and infection risk.

***In vivo toxicity and biodegradability evaluation***

*In vivo* toxicity and hydrogel biodegradability were monitored over a 3-month observational period following surgical procedures, adhering to UNIENISO10993-6 guidelines. Every two weeks during the experiment, parameters of general integrity were evaluated using a modified SHIRPA protocol ^[4]^. During the experimental period, body weight was monitored and normalized relative to the pre-implant weight. The modified SHIRPA protocol included the observation and scoring of macro-parameters such as activity, tremor, lacrimation, eyelid closure, fur appearance, whisker movement, and defecation, with 0 = absent or 1 = present. Locomotion and anxiety, which are early indicators of systemic toxicity ^[5,6]^, were evaluated using the open field (OF) test once a month for 3 months. Behaviours were recorded and automatically analyzed using the ANY-maze Video Tracking system (Stoelting). Motor activity and exploration in a new environment were assessed by placing mice in the centre of an open wooden chamber (50×50×40 cm) with dark walls and allowing them to explore freely for 10 minutes. The space was virtually divided into 3 zones (periphery, centre, and corners) for indirect assessment of anxiety. Distance travelled, maximum speed, and time spent in each zone were automatically recorded. Time spent in the centre was considered an anxiety index (anxious mice tend to spend more time in the periphery). The apparatus was cleaned with 70% ethanol after each test to eliminate olfactory cues. Hydrogel biodegradability was verified at sacrifice by inspecting the skin of the implantation site to visually detect any remaining material.

**Ex vivo toxicity evaluation**

***Sacrifice, blood, and organ collection***

At the end of the experimental period, mice were sacrificed under general anesthesia with inhaled isoflurane. Blood was collected, allowed to coagulate at room temperature for 40 minutes, and centrifuged at 1200 g for 15 minutes to extract serum for hepatic marker evaluation. Organs (liver, spleen, kidneys, heart, lungs, and brain) were collected and weighed to assess any macroscopic alteration. A sample of back skin above the implant area was also collected.

***Hepatic toxicity evaluation***

An important indicator of systemic toxicity is impaired liver function. Serum Alanine Transaminase (ALT) activity (U/L) was evaluated using an enzymatic colorimetric assay according to the manufacturer’s instructions (Sigma-Aldrich). ALT activity was measured using 20 µl of serum per sample, with absorbance read at 570 nm (Multiskan FC, Thermo Scientific) and calculated using the linear regression method with a pyruvate standard curve.

***Histological Analysis of Skin***

Post-fixed skin samples were post-fixed in 4% paraformaldehyde for 72 hours and immersed in 20% sucrose-PBS for 2 days, followed by 30% sucrose in PBS for 3 additional days. The tissues were embedded in histo-mount medium using dry ice and stored at −80 °C until further processing. Cryosections (40 μm thick) were obtained using a Leica CM1520 cryostat at −27 °C and mounted onto slides, which were subsequently stored at −20 °C until use. Prior to histological staining, the slides were air-dried at room temperature for 4 hours. Hematoxylin and eosin (H&E) staining was performed according to validated protocols ^[7]^. Histological images were acquired using an optical microscope (Nikon Eclipse Ci) equipped with a 10× objective. The thickness of individual skin layers (granular layer, epidermis and dermis, hypodermis, and muscle layer) was quantified using the length measurement tool of the NIS-Elements software (Nikon).

***On demand In vivo Hydrogel Degradation***

The hydrogel was brought to the solid form by staying in an oven at 37 °C. Subsequently, 1 g of solid hydrogel was implanted in the back of mice without suturing the pocket to visually check the gel-sol transition. An ice cube was applied in contact with the skin of the pocket (T ~ 25 °C) for a maximum time of 3 minutes. In a second group of mice (Control), 1 g of solid hydrogel was implanted in the same way without applying the temperature variation to observe the maintenance of the solid state in 3 minutes of observation.

**Table S1.** Gelation temperature (Tgel), melting temperature (Tm), and gelation time (tgel) at 37 °C for the developed hydrogels (error calculated to ± 5° phase angle).

| **Formulation** | **Tgel (°C)** | **Tm (°C)** | **tgel (s)** |
| --- | --- | --- | --- |
| MC/PEG | 30.6 ± 2.8 | 7.7 ± 0.4 | 70 ± 30 |
| MC/PEG/PAA | 43.6 ± 0.6 | 2.9 ± 0.4 | 60 ± 20 |
| MC/PEG/PEGDA | n.d. | n.d. | n.d. |
| MC/PEG/PEGMA | n.d. | n.d. | n.d. |

**Table S2. Statistical analysis.** Statistical analysis of mice during the 12 weeks of observation. * p ≤ 0.05, ** p ≤ 0.01, *** p ≤ 0.001

| **Analyzed parameter** | | **Statistical test** | **Variable** | **Df,F** | **P value** | **Signific.** |
| --- | --- | --- | --- | --- | --- | --- |
| Weight (%) | | Two-way repeated measure ANOVA (sex as covariate) | Type of implant | (1,8) = 0.077 | 0.789 | Ns |
|  |  |  | Sex | (1,8) = 0.139 | 0.719 | Ns |
|  |  |  | Time | (8,8) = 8.032 | < 0.001 | *** |
|  |  |  | Time*Type of implant | (8,8) = 0.079 | 1 | Ns |
| Maximum speed (m/s) | | Two-way repeated measure ANOVA (sex as covariate) | Type of implant | (1,8) = 2.594 | 0.146 | Ns |
|  |  |  | Sex | (1,8) = 5.551 | 0.046 | * |
|  |  |  | Time | (3,24) = 0.693 | 0.565 | Ns |
|  |  |  | Time*Type of implant | (3,24) = 3.607 | 0.028 | * |
| Total distance travelled (%) | | Two-way repeated measure ANOVA (sex as covariate) | Type of implant | (1,8) = 2.276 | 0.170 | Ns |
|  |  |  | Sex | (1,8) = 1.642 | 0.236 | Ns |
|  |  |  | Time | (3,8) = 1.258 | 0.311 | Ns |
|  |  |  | Time*Type of implant | (3,8) = 5.144 | 0.007 | ** |
| **Time spent in the centre (%)** | | Two-way repeated measure ANOVA (sex as covariate) | Type of implant | (1,8) = 0.362 | 0.564 | Ns |
|  |  |  | Sex | (1,8) = 2.264 | 0.171 | Ns |
|  |  |  | Time | (3,24) = 0.974 | 0.421 | Ns |
|  |  |  | Time*Type of implant | (3,24) = 0.437 | 0.728 | Ns |
| Organ weight normalized over body weight (%) | | Two-way repeated measure ANOVA (sex as covariate) | Type of implant | (1,8) = 0.011 | 0.921 | Ns |
|  |  |  | Sex | (1,8) = 2.610 | 0.145 | Ns |
|  |  |  | Organ type | (5,8) = 27.915 | <0.001 | *** |
|  |  |  | Organ type*Type of implant | (5,8) = 0.083 | 0.995 | Ns |
| ALT activity (U/L) | | Univariate test (sex as covariate) | Type of implant | (1,9) = 0.160 | 0.701 | Ns |
|  |  |  | Sex | (1,9) = 0.970 | 0.35 | Ns |
| Histological analysis of skin | Total thickness (µm) | Univariate ANOVA | Type of implant | (1,10) = 0.150 | 0.707 | Ns |
|  | Thickness (µm) of individual skin layers | Two-way repeated measure ANOVA | Type of implant | (1,9) = 0.158 | 0.700 | Ns |
|  |  |  | Skin layers | (3,9) = 30.249 | < 0.001 | *** |
|  |  |  | Skin layers*Type of implant | (3,9) = 0.136 | 0.938 | Ns |


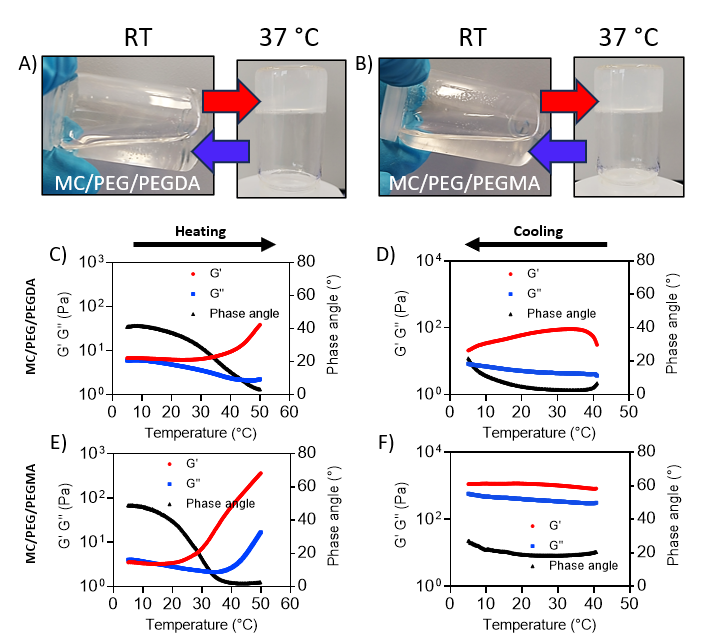


**Figure S1. Inversion tube test of the MC/PEG/PEGDA (A) and MC/PEG/PEGMA (B) hydrogels.** Pictures are taken immediately after the mixing (left) and after incubation at 37 °C (right). **Temperature-dependent rheological analysis of MC/PEG/PEGDA (C-D) and MC/PEG/PEGMA (E-F) hydrogels.** Samples were heated from 5 °C to 50 °C at 1 °C/min. Frequency was 1 Hz and shear strain 3%. Tgel is defined as the crossover of storage G’ and loss modulus G^’’^: (C) MC/PEG/PEGDA and (E) MC/PEG/PEGMA. To evaluate gel-sol transition, the samples were initially at their gel state (at 37 °C) and then were cooled from 40 °C to 5 °C at 1 °C/min. Frequency was 1 Hz and shear strain 3%. Tm is defined as the crossover of storage G' and loss modulus G'': MC/PEG/PEGDA (D) and MC/PEG/PEGMA (F).


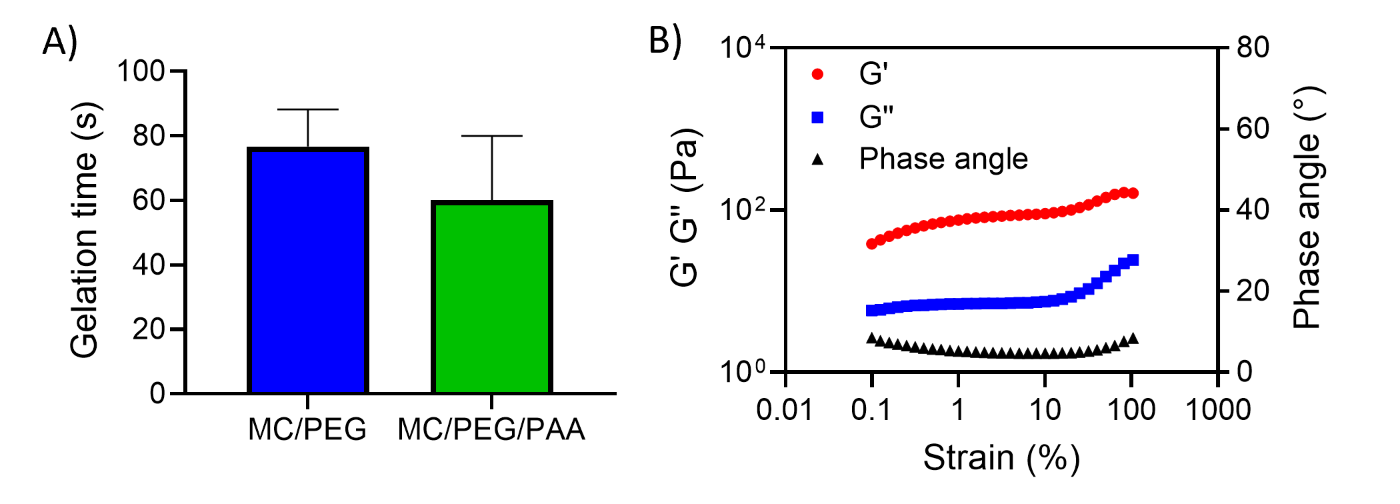


**Figure S2. (A) Average gelation time at 37 °C for MC/PEG and MC/PEG/PAA hydrogels.** Each point represents the mean ± SD (n = 3). **(B)** **Strain amplitude sweep of the MC/PEG/PAA hydrogel performed at 37 °C** with a constant angular frequency of 1 Hz. Strain amplitude was increased from 0.1% to 100% to evaluate the linear viscoelastic region (LVER) and mechanical stability of the formulation. The storage modulus (G′), loss modulus (G″), and phase angle were recorded as functions of strain.


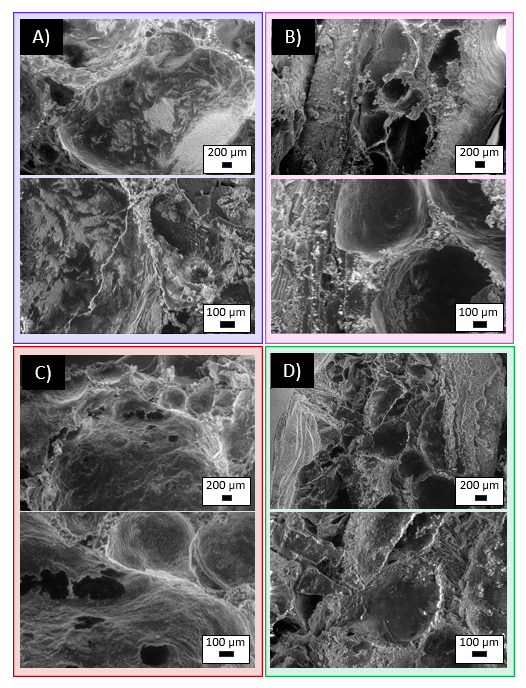


**Figure S3. Cross-section SEM images of hydrogel MC/PEG (A), MC/PEG/PEGDA (B), MC/PEG/PEGMA (C), and MC/PEG/PAA (D).** Magnification 60X (top) and 200X (bottom).

**
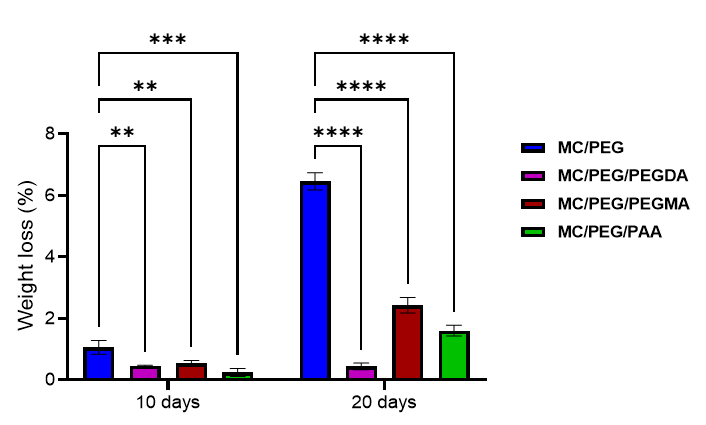
**

**Figure S4. Weight loss (%) of MC/PEG, MC/PEG/PEGDA, MC/PEG/PEGMA, and MC/PEG/PAA hydrogels after 10 and 20 days of incubation in PBS (pH 7.4).** Data are presented as mean ± standard deviation (n = 3). Statistical significance was assessed by two-way ANOVA with Dunnett's multiple comparison test. Asterisks indicate statistically significant differences between conditions (** p < 0.01,*** p < 0.001, **** p < 0.0001). Multiple comparison test at 10 days showed significant differences between MC/PEG and MC/PEG/PEGDA (p = 0.0025), MC/PEG and MC/PEG/PEGMA (p = 0.0078), and MC/PEG and MC/PEG/PAA (p = 0.0002). At 20 days, statistically significant differences were observed between MC/PEG and MC/PEG/PEGDA (p < 0.0001), MC/PEG and MC/PEG/PEGMA (p < 0.0001), and MC/PEG and MC/PEG/PAA (p < 0.0001).

**
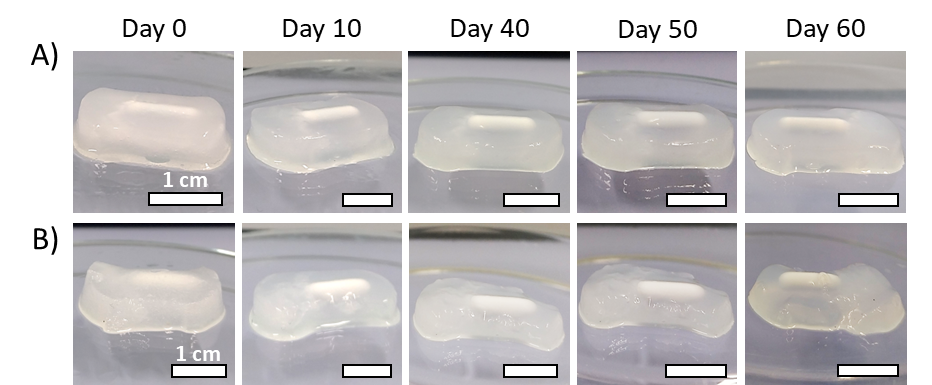
**

**Figure S5. Representative images of the degradation of the hydrogels MC/PEG/PEGDA (A), MC/PEG/PEGMA (B). An inert magnet was encapsulated in the polymer matrix, as a surrogate model for simulating the incorporation of an implantable device. Samples were incubated in PBS buffer (pH 7.4) at 37 °C to evaluate morphological and structural stability at 0, 10, 40, 50 and 60 days.**


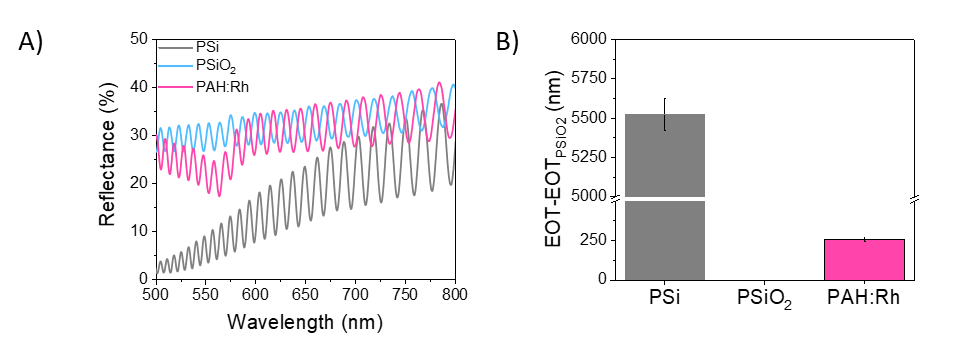


**Figure S6. Optical reflectance measurements on the fluorescent device used as a model sensor to encapsulate within MC/PEG/PAA hydrogel. (A)** Reflectance spectra recorded in air on a PSi scaffold and PSi oxidized at 1000 °C for 5 min (PSiO_2_) before and after the functionalization with PAH:Rh. **(B)** EOT-EOT_PSiO2_ values recorded for PSi scaffold both as-prepared and after functionalization with PAH:Rh. The EOT value of oxidized PSi interferometers (i.e., EOT_PSiO2_) is used as reference to obtain positive differential EOT values. Data are reported as mean values from three independent devices, with error bars representing standard deviation (SD).


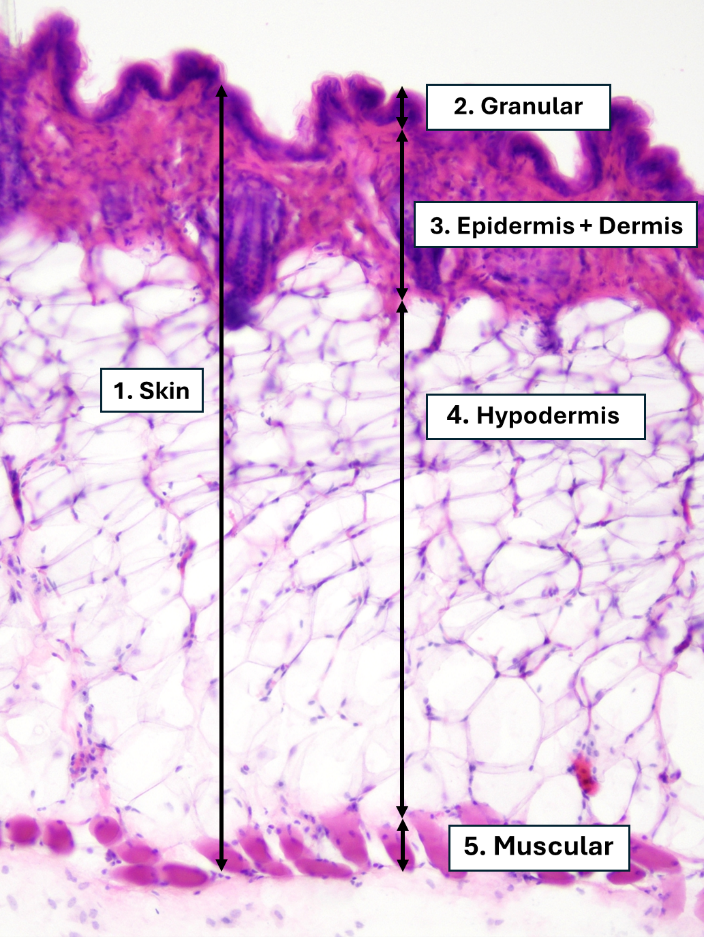


**Figure S7.** Histological image of skin with its different layers. Layers: 1) Skin, 2) Granular, 3) Epidermis + Dermis, 4) Hypodermis, and 5) Muscular.

**
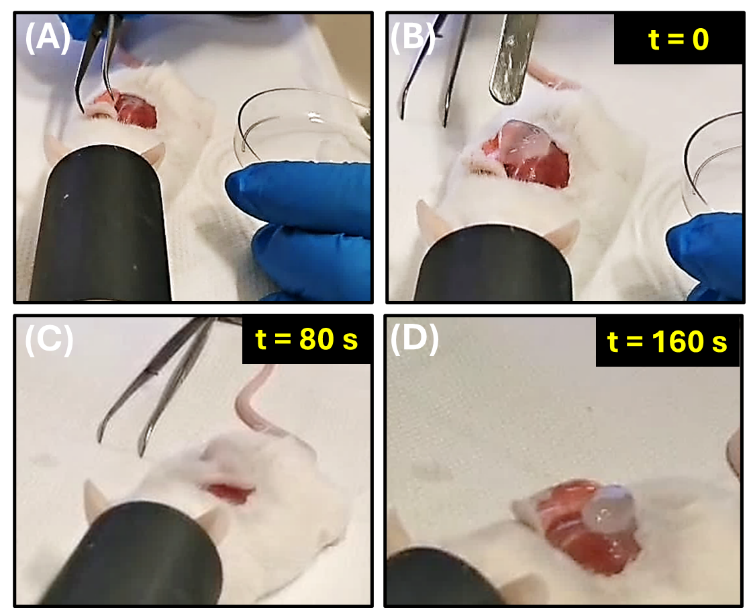
**

**Figure S8. In vivo evaluation of MC/PEG/PAA hydrogel stability in control animals without thermal input.** The hydrogel is implanted in the back of mice without suturing the pocket to visually check its status **(A)**. The time-lapse at different time intervals **(B-D)** show that the implanted hydrogel remains in the gel state when no external thermal stimulus is applied, demonstrating its stability at physiological conditions.

**References**

[1] L. Lamanna, F. Rizzi, C. Demitri, M. Pisanello, E. Scarpa, A. Qualtieri, A. Sannino, M. De Vittorio, *Cellulose* **2018**, *25*, 4331.

[2] M. R. Martínez-Miranda, V. García-Martínez, M. R. Gude, *Polym Test* **2019**, *78*, 105950.

[3] M. J. Sailor, *Porous Silicon in Practice: Preparation, Characterization and Applications* **2012**.

[4] D. C. Rogers, J. Peters, J. E. Martin, S. Ball, S. J. Nicholson, A. S. Witherden, M. Hafezparast, J. Latcham, T. L. Robinson, C. A. Quilter, E. M. C. Fisher, *Neurosci Lett* **2001**, *306*, 89.

[5] A. K. Kraeuter, P. C. Guest, Z. Sarnyai, *Methods in Molecular Biology* **2019**, *1916*, 99.

[6] E. Daini, S. Hagmeyer, C. A. De Benedictis, J. S. Cristóvão, M. Bodria, A. M. Ross, A. Raab, T. M. Boeckers, J. Feldmann, C. M. Gomes, M. Zoli, A. Vilella, A. M. Grabrucker, *Translational Psychiatry 2021 11:1* **2021**, *11*, 1.

[7] M. Corsi, A. Paghi, S. Mariani, G. Golinelli, A. Debrassi, G. Egri, G. Leo, E. Vandini, A. Vilella, L. Dähne, D. Giuliani, G. Barillaro, *Advanced Science* **2022**, *9*, 2202062.
